# A review on application of nanoparticles for EOR purposes: history and current challenges

**DOI:** 10.1007/s13202-022-01606-x

**Published:** 2023-01-10

**Authors:** Mostafa Iravani, Zahra Khalilnezhad, Ali Khalilnezhad

**Affiliations:** 1 Faculty of Petroleum and Natural Gas Engineering, Sahand University of Technology, Tabriz, 51335-1996 Iran; 2 Department of Economics, Shiraz University, Shiraz, Iran; 3 Grupo de Investigación en Fenómenos de Superficie−Michael Polanyi, Facultad de Minas, Universidad Nacional de Colombia, Sede Medellín, 050034 Medellín, Colombia

**Keywords:** Nanoparticles, EOR, Low salinity, Polymer, Economy

## Abstract

Applications of nanotechnology in several fields of petroleum industry, e.g., refinery, drilling and enhanced oil recovery (EOR), have attracted a lot of attention, recently. This research investigates the applications of nanoparticles in EOR process. The potential of various nanoparticles, in hybrid and bare forms for altering the state of wettability, reducing the interfacial tension (IFT), changing the viscosity and activation of other EOR mechanisms are studied based on recent findings. Focusing on EOR, hybrid applications of nanoparticles with surfactants, polymers, low-salinity phases and foams are discussed and their synergistic effects are evaluated. Also, activated EOR mechanisms are defined and specified. Since the stabilization of nanofluids in harsh conditions of reservoir is vital for EOR applications, different methods for stabilizing nanofluids through EOR procedures are reviewed. Besides, a discussion on different functional groups of NPs is represented. Later, an economic model for evaluation of EOR process is examined and “Hotelling” method as an appropriate model for investigation of economic aspects of EOR process is introduced in detail. The findings of this study can lead to better understanding of fundamental basis about efficiency of nanoparticles in EOR process, activated EOR mechanisms during application of nanoparticles, selection of appropriate nanoparticles, the methods of stabilizing and economic evaluation for EOR process with respect to costs and outcomes.

## Introduction

Nanotechnology as a pioneering field of knowledge has prevailed various branches of science. High surface area-to-mass ratio, small size of nanoparticles, special chemical and physical properties and various morphology of particles are some positive points about nanotechnology (Sabet et al. 2016). Petroleum engineering like other fields of industry should become updated with respect to advances of science and technology. Capability of nanoparticles for EOR intends is an interesting topic for EOR researchers and experts. On average, 30–50% of original oil reserve is producible by natural mechanisms in reservoirs. High amount of remaining oil illustrates the key role of EOR procedures for gaining the maximum possible income from an oil reservoir. Pressure maintenance, improving mobility of reservoir fluids and producing the trapped oil are known as the main goals of EOR procedures. Usually, water and gas injection are initial EOR process. These operations are named secondary methods and performed to maintain the pressure of reservoirs (Sheng 2010). Considering the condition of reservoir and amount of trapped oil after water or gas injection, chemical agents or low-salinity water could be injected into reservoirs. Since these methods are used after water or gas injection, they are named as tertiary methods or chemical EOR methods (cEOR) (Sheng 2010). Chemical EOR methods are mostly used to reduce interfacial tension, alter the state of wettability and improve sweep efficiency by mobility control (Gbadamosi et al. 2019b). IFT is an important factor for obtaining miscible displacement. Lower values of IFT is desired for miscible displacement. On the other hand, natural wettability of reservoir rocks is usually oil wet. To achieve more amount of oil, water-wet and neutral wet conditions are preferred. In addition, early breakthrough due to viscous fingering is a restriction for EOR methods (Khalilnezhad et al. 2021). Some cEOR methods are used to prevent this phenomenon by mobility control. Polymer flooding, surfactant flooding, foam flooding and injection of low-salinity phase are the most common cEOR methods.

Polymers are used to avoid viscous fingering and improve sweep efficiency (Sorbie 2013). Due to high viscosity, they are capable to control the mobility of fluids (Xiangguo et al. 2021). Several studies and field applications confirmed the efficiency of polymers for the enhancement of oil recovery (De-Min et al. 2005; Han et al. 2006; Mishra et al. 2014; Wang et al. 2009).

As mentioned before, low IFT values are desirable for EOR process. Surfactants by taking advantage of their nonpolar heads and polar tails are appropriate agents for reducing IFT (Belhaj et al. 2020). Besides, utilization of surfactants along with high gas contents results in foam generation. Foams have higher viscosity than gas and can improve sweep efficiency compared to gases (Hosseini-Nasab and Zitha 2017).

**Fig. 1:**
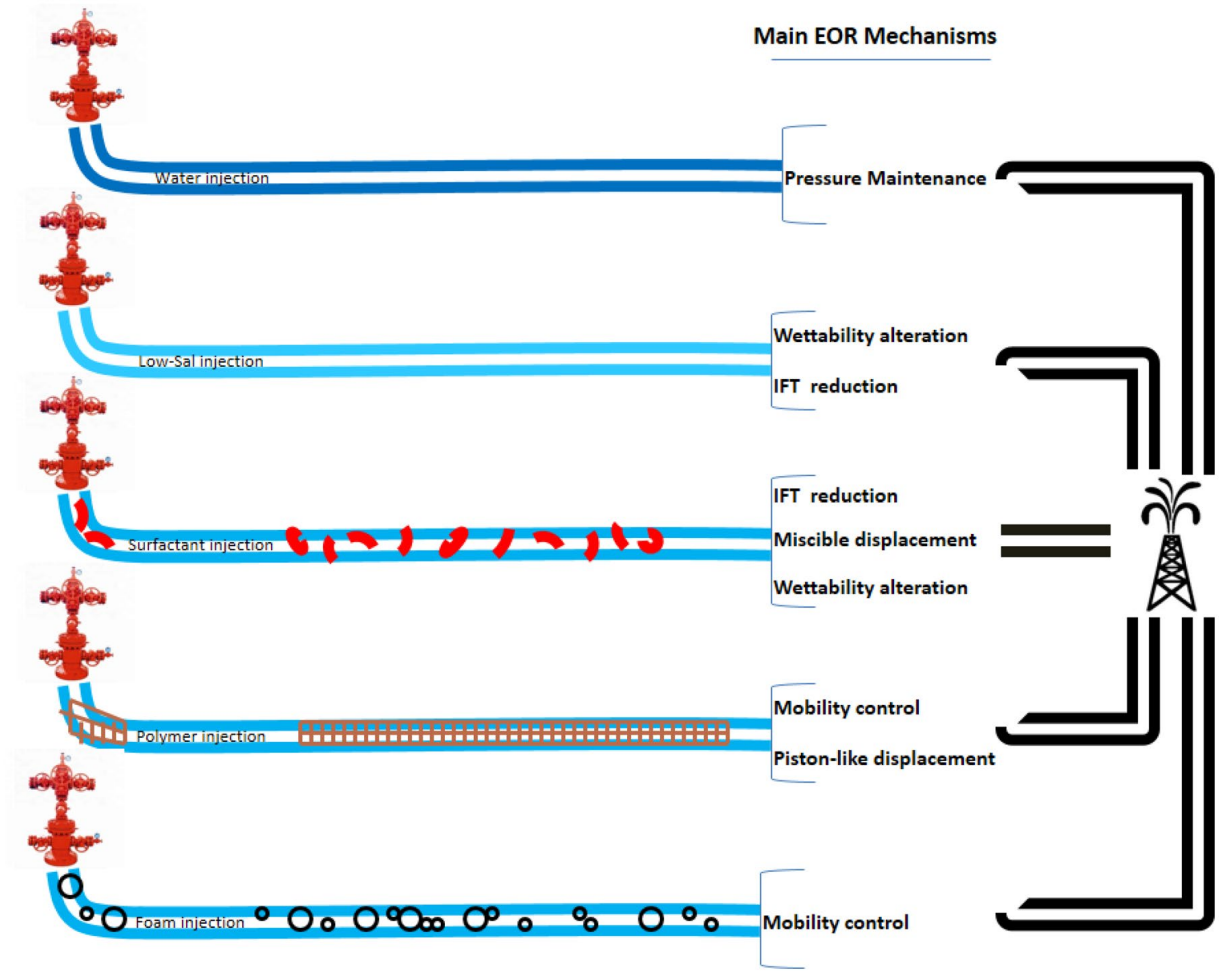
Schematic view of different cEOR methods and their main mechanism

Injection of low-salinity water into reservoirs is proved as an efficient EOR method (Lyu et al. 2022; Sheng 2014). Wettability alteration is the main activated mechanism by this method (Liu and Wang 2020). Figure 1 presents the mentioned cEOR methods and reported mechanisms for them.

There is some special conditions and limitations in EOR process which could be mitigated by nanotechnology. Although application of nanoparticles for the enhancement of oil recovery is faced with some uncertainties, several pilot tests and field applications are reported all around the world (Franco et al. 2021; Huang et al. 2010; Kaito et al. 2022; Kanj et al. 2011). Maintaining the stability of nanofluids during injection into reservoirs and selecting the proper size for nanoparticles to avoid pore blockage, economic feasibility and compatibility of selected NPs with production severities are the main challenges for the application of nanoparticles in EOR process which are discussed in the following sections. Tolerating harsh condition of reservoirs, catalytic effects, tendency of wettability alteration, locating at the interface of immiscible fluids, etc. made nanoparticles appropriate candidates for application in reservoirs. Recently, hybrid application of nanoparticles with chemical agents used in EOR process is widely investigated. Taking the advantages of nanofluids and other chemical agents like polymers and surfactants is the main goal of these studies. Most of the obtained results reported fair capability for NPs to empower EOR mechanisms.

Studying EOR mechanisms and synergistic effects of applications of nanoparticles for EOR intends, this review includes numerous recent researches on nanotechnology. Focusing on applications of nanoparticles and activated EOR mechanisms, critical parameters, functional groups of nanoparticles and methods of stabilizing nanofluids, representing an economic model for determination of incomes and costs and categorizing the studies due to applied nanoparticles are important points which distinguishes this article from others with the same subjects. Although synthesizing nanoparticles and environmental challenges are not covered because of specified capacity of this work, these subjects could be evaluated in further investigations. In this research, first the activated EOR mechanisms by bare nanoparticles are discussed. Then the importance of the size of nanoparticles and its related advantages and disadvantages are investigated. Numerous researches based on performed analysis, applied nanoparticles and reported EOR mechanisms are tabulated in this section. Then the functional groups of some nanoparticles which are frequently used for EOR process are introduced. Thereafter, methods of stabilizing nanofluids are investigated. Sequentially, hybrid applications of nanoparticles and surfactants, polymers, low-salinity phases and foams are evaluated based on literature reviews. Finally, “Hotelling” method is introduced for economic evaluation of EOR process.

### Nanoparticle mechanisms for EOR purposes

NPs could be used in petroleum industry for different goals such as enhancement of oil recovery, improved drilling and exploration (tracers). In this section, the effects of NPs on the enhancement of oil recovery is investigated. Besides, the introduced mechanisms of NPs and some of the last obtained experimental results are presented.

#### Effects of NPs on rock and fluid system

Several studies have introduced NPs as an effective agent for changing properties of rock and fluid system. Many researchers reported achievement of greater amounts of oil during application of NPs (Sun et al. 2017). To seek the effects of nanoparticles concentration on wettability alteration, Huibers et al. dispersed different amounts of silica nanoparticles in brine and checked their efficiency in 2 different sandstones (Berea and Boise). They concluded that the presence of silica nanoparticles in the brine causes wettability alteration. Also, they observed a linear correlation between the concentration of nanoparticles and wettability alteration (Huibers et al. 2017).

Since the oil film which has covered the surface of rock might contain palmitic acid, Hou et al. examined the performance of silica nanoparticles in the presence of sodium. They reported that the hydrophilic silica NPs are capable of altering the wettability of carbonate rocks by adsorption to calcite surfaces. Moreover, they found that there is a synergistic effect for Na^+^ ions and silica nanoparticles in wettability alteration. As a matter of fact, since sodium cation is able to compress electric double layer and neutralize the negatively charged surfaces of rock, it raises the chance of silica NPs to being adsorbed by the rock surface in competition with palmitic acid content of oil (Hou et al. 2019). Usually, the efficiency of NPs on wettability alteration is evaluated at ambient conditions. It is obvious that the reservoir condition differs from ambient condition. Al-Anssari et al. investigated the efficiency of silica nanoparticles at reservoir condition in the presence of sodium dodecyl sulfate (SDS) surfactant. They realized that the wettability of carbonate rock could be altered from strongly oil wet to water wet using surfactant–NPs suspensions (at 70 °C and 20 MPa) (Al-Anssari et al. 2017).

**Fig. 2:**
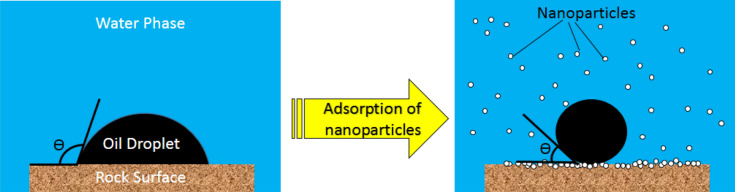
Wettability alteration due to adsorption of nanoparticles on the surface of rock

Khalilnezhad et al. investigated the effects of titania NPs on wettability alteration. They observed that 1000 ppm concentration of titania induces the greatest wettability alteration to their carbonate rock. They reported precipitation and adsorption of NPs on the surface of rock as the main mechanism of wettability alteration (Khalilnezhad et al. 2019). Rezvani et al. compared wettability alteration of a carbonate rock by MgO, SiO_2_, Fe_3_O_4_ and ZnO nanoparticles. They introduced silica as the best wettability modifier among others. In addition, Fe_3_O_4_ NPs reflected the weakest response for wettability alteration (Rezvani et al. 2018a, b, c). Adsorption of NPs on the surface of rocks takes place by several mechanisms. NPs could be adsorbed to the surface of rock due to surface charges. Calcite content of carbonate rocks has positive charge in the presence of water. NPs with negative charge will be adsorbed to the surface of rock with respect to electrostatic attraction. Besides, agglomeration of NPs results in precipitation on the surface of rock by gravity force. As Dehghan Monfared et al. claimed, gradual release of carboxylate group from surface of rock and substitution by NPs is a governing mechanism for wettability alteration in oil-wet rocks (Dehghan Monfared et al. 2016). In addition, smaller size of particles results in high disjoining pressure due to great repulsion between NPs. Therefore, adsorption and precipitation will be intensified for smaller size of particles. Figure 2 presents alteration of wettability due to application of NPs schematically. As it could be observed, adsorption of NPs on the surface of rock creates a new surface and reduces the contact angle of water significantly.

#### Efficiency of NPs on fluid–fluid interaction and interfacial tension (IFT)

The reduction in the IFT is a vital mechanism for achievement of miscibility and increases the efficacy of water flooding process. IFT is usually affected by some parameters like salinity, pH, asphaltene content of crude oil, etc. (Behrang et al. 2021). There are numerous evident that prove NPs are capable of reducing the interfacial tension (IFT) of oil and water. Hydrophobicity and hydrophilicity of NPs play key roles in attachment of NPs to the interface of immiscible fluids. Equation 1 describes the dependence of adhesion energy on contact angle. This equation could be used to investigate the behavior of NPs at the interface of fluids (Ngai and Bon 2014).

1 $$\Delta E = \pi R^{2} \sigma_{12} \left( {1 \pm {\text{Cos}} \theta_{12} } \right)^{2}$$

where *E* represents adhesion energy (K_B_T), *R* is the radius of particle (nm), ${\sigma }_{12}$ defines the interfacial tension between 2 fluid phases (mNm^−1^) and $\theta$ is the contact angle of particle at the surface of fluids. In fact, the required energy for detachment of NPs from interface could be calculated by this equation. Obviously, the magnitude of adhesion energy for smaller particles is lower than greater ones. Therefore, the interfacial attachment of smaller particles is less than larger ones.

Hosseini et al. examined the effects of NPs concentration in the range of 0.01–5 wt% on IFT. Finally, they concluded that increasing the concentration of nanoparticles decreases IFT. Also they expressed that NPs could decrease the value of IFT about 50%. However, this value is not as high to the extent that be considered as a significant EOR mechanism (Hosseini et al. 2019). Rezvani et al. checked out the potential of ZrO_2_ NPs for application in EOR process at reservoir conditions. They observed that the addition of zirconium oxide NPs to the diluted formation water reduces IFT. Also, by observing the behavior of various concentrations they claimed that there is an optimum concentration for zirconium oxide NPs. Further addition of NPs for obtaining nanofluids above the optimum concentration causes an inverse trend, and IFT increases directly with any increase in concentration (Rezvani et al. 2018a).

Some studies assessed the synergic effects of NPs with other chemicals used for EOR procedures. Betancur et al. designed a core shell system for iron NPs. They obtained the lowest amount of IFT (1 × 10^–4^ mNm^–1^) with the addition of NPs to the surfactant mixture. This ultralow value achieved as a result of reduced adsorption of surfactant mixture on the surface of porous media. Coated NPs diminished the adsorption by a rate of approximately 33% (Betancur et al. 2019). In another study, Al-Anssari et al. used hydrophilic and hydrophobic silica NPs to investigate their influence on IFT in CO_2_/brine systems. Their observations proved that the pressure and the concentration of NPs have positive effects on IFT reduction, but temperature and salinity have negative effects. The results indicated the potentials of using NPs with carbonated water for the enhancement of oil recovery (Al-Anssari et al. 2018b). It could be concluded same as surfactants (Alabdulbari et al. 2022), NPs are also capable of reducing IFT of CO_2_/brine system. Surfactants have some restricting factors like salinity, ion types (monovalent, divalent, etc.), temperature and surfactant type (anionic, cationic and nonionic) (Golabi et al. 2012).

IFT as a thermodynamic property changes by time. Variation of IFT is governed by mass transfer between oil and water. The more mass transfer between oil and water leads to lower IFT values. Same as surfactants, some NPs have both hydrophilic and hydrophobic parts simultaneously which facilitates the movement of NPs in the bulk phase and their attachment onto the interface of fluids. The tendency of NPs for attachment to the interface of fluids, on the one hand, and their catalytic effect in asphaltene adsorption, on the other hand, are the main reasons for forming a layer between oil and water. Due to their tendency for adsorption of asphaltenes, mass transfer between fluids increases in the presence of NPs and sequentially IFT decreases.

#### Other effective mechanisms

Not only NPs are capable of altering the state of wettability and reducing the value of IFT, but also they can activate some other EOR mechanisms in different situations. Some NPs have the tendency to reduce the viscosity of oil by preventing asphaltene precipitation and cracking the long chains. Patel et al. examined the effects of 3 metal oxide NPs on reducing the viscosity of a sample of heavy oil. They observed that all tested concentrations of NiO, CuO and Fe_3_O_4_ can reduce the viscosity of heavy oil more than 50% (Patel et al. 2018). Elshawaf et al. investigated the effects of different types of NPs on lowering the viscosity of a heavy asphaltic oil. They observed the highest reduction (20–65%) during application of graphene oxide. Moreover, they reported that graphene oxide is more valuable from economic point of view (Elshawaf 2018). Taborda et al. also studied the effects of Fe_3_O_4_, SiO_2_ and Al_2_O_3_ NPs on reducing viscosity of heavy and extra heavy crudes. They observed SiO_2_ at concentration of 1000 mg/l reduces the viscosity around 52% which is the best result among all. Finally they matched their experimental results with Pal and Rhodes model (Eq. 2) (Pal and Rhodes 1989). This model correlated the viscosity with the concentration of NPs.

2 $$\mu_{r} = \left( {1 + K_{0} C} \right)^{V}$$

where ${\mu }_{r}$ is the ratio of dispersion viscosity to bulk phase viscosity; *K*_0_ represents the solvation constant; *C* defines volumetric concentration of NPs (W/Vol); and *V* is the shape factor of dispersed particle. The model covered their experimental results as well (Taborda et al. 2017).

In another study, Ghaffari et al. synthesized a colloidal gel by silica NPs. They reported high concentrations of silica NPs (in the range of 3–6 wt%) at high-salinity conditions facilitate the formation of a viscous gel which could be used in both EOR and water shutoff projects. The reason of forming this gel is entrapment of water clusters between silica NPs. In fact, the presence of salt leads to agglomeration of silica NPs and agglomerated NPs entrap the water ganglia (Ghaffari et al. 2022). The main mechanism for reducing viscosity of heavy oil by NPs is adsorption and cracking of heavy compounds and consequently diminishing the size of aggregations. Therefore, NPs are capable of reducing the viscosity of heavy oils.

### Size as a critical parameter for selection of NPs

**Fig. 3:**
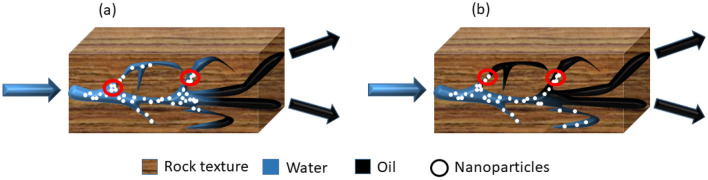
Schematic illustration of log jamming (**a**) and pore plugging (**b**)

The existence of tight and tortuous paths in porous media is a challenging factor for application of the nanoparticles. Mean free path and pore size distribution are two vital properties which should be considered before selection of any nanoparticles for EOR procedure (Collins 1976; Dullien 2012). If the radius of nanoparticles be greater than the pore throats, pore plugging or log jamming will be inevitable. Both mentioned phenomena are mechanistically similar, but they differ in results. Both of them refer to plugging the pores by nanoparticles, but when log jamming takes place nanoparticles will plug the paths of swept zones and the flow will be diverted to the upswept areas of reservoir. Therefore, log jamming is a positive mechanism for the enhancement of oil recovery (El-Diasty and Aly 2015). In contrast, when pore plugging phenomenon occurs, aggregation of nanoparticles in the entrance of upswept paths makes them unreachable and consequently lowers the expected oil recovery (Ju et al. 2006) which is a common phenomenon in the cases of tight reservoirs. Nanoparticles should be injected in very low concentrations in this type of reservoirs (Lu et al. 2017). Figure 3 illustrates the differences between log jamming and pore plugging.

By analyzing 20 core plugs with a mixture of surfactant and silica nanoparticles, Rezaei et al. introduced pore size distribution as one of the most important parameters for nanofluid injection (Rezaei et al. 2020). Jiang et al. investigated the effects of size of bare silica NPs on wettability alteration and oil recovery of carbonate rocks. They checked different sizes (10, 40, 90 and 150 nm) of silica NPs. They concluded that the smaller NPs intensify the alteration of wettability. Hence, the amount of oil recovery was greater through application of smaller NPs (Jiang et al. 2017). Some important studies (since 2017) on application of bare nanoparticles and nanocomposites for enhancing oil recovery are tabulated in Table 1.

**Table 1:** Some studies on application of nanoparticles and nanocomposites in EOR

No	Title	NPs	Analyses	EOR mechanism(s)
1	Experimental investigation of silica-based nanofluid enhanced oil recovery: the effect of wettability alteration (Li et al. 2017)	Silica	Micromodel injection Spontaneous inhibitions	Wettability alteration
2	Potential effects of metal oxide/SiO_2_ nanocomposites in EOR processes at different pressures (Kazemzadeh et al. 2018)	(nanocomposites) TiO_2_/SiO_2_ Fe_3_O_4_/SiO_2_	Contact angle IFT measurement at high pressure Viscosity measurement Adsorption of asphaltene	Wettability alteration IFT reduction Lowering viscosity Asphaltene adsorption
3	Wettability of nanofluid-modified oil-wet calcite at reservoir conditions (Al-Anssari et al. 2018a)	Hydrophilic silica	Contact angle measurement at high pressure–temperature and salinity	Wettability alteration
4	Reversible and irreversible adsorption of bare and hybrid silica nanoparticles onto carbonate surface at reservoir condition (Al-Anssari et al. 2020a)	Hydrophilic and functional silica	DLS SEM AFM EDS	Attachment of NPs on surface of calcite (wettability alteration)
5	New insights into application of nanoparticles for water-based enhanced oil recovery in carbonate reservoirs (Gomari et al. 2019)	Silica Al_2_O_3_	Zeta potential measurement Contact angle pH measurement (at different salinity and pressure conditions)	Wettability alteration
6	Wettability Alteration in Carbonate Reservoirs by Carbon Nanofluids (Kanj et al. 2020)	Carbon nanodots	Static and dynamic contact angle Zeta potential Spreading on surface	Wettability alteration
7	Effect of Nanoparticles on the Interfacial Tension of CO2-Oil System at High Pressure and Temperature: An Experimental Approach (Al-Anssari et al. 2020b)	Hydrophilic and hydrophobic silica	Measurement of IFT at elevated pressure and temperature	IFT reduction
8	A Mechanism Study of Wettability and Interfacial Tension for EOR Using Silica Nanoparticles (Jiang et al. 2017)	Silica	Contact angle measurement on quartz IFT measurement Core flood	Wettability alteration IFT reduction
9	The effect of nanoparticles on wettability alteration for enhanced oil recovery: micromodel experimental studies and CFD simulation (Rostami et al. 2019)	Silica	Contact angle measurement Micromodel injection CFD simulation	Wettability alteration
10	Silica-based amphiphilic Janus nanofluid with improved interfacial properties for enhanced oil recovery (Wu et al. 2020)	Janus silica	Contact angle measurement IFT measurement Interfacial viscosity measurement Core flood	Wettability alteration IFT reduction Mobility control (Increase in interfacial shear viscosity)
11	Application of functionalized silica-graphene nanohybrid for the enhanced oil recovery performance (Tajik et al. 2018)	Silica/graphene	IFT measurement Visual stability of emulsions Micromodel injection	IFT reduction
12	Wettability modification of oil-wet carbonate reservoirs using silica-based nanofluid: An experimental approach (Aghajanzadeh et al. 2019)	Silica	Contact angle measurement for various salinities and rock types Imbibition Core flood	Wettability alteration
13	Effect of NiO/SiO2 nanofluids on the ultra-interfacial tension reduction between heavy oil and aqueous solution and their use for wettability alteration of carbonate rocks (Dahkaee et al. 2019)	NiO, silica and NiO/SiO_2_ (nanocomposites)	IFT measurement (with and without preheating of nanofluid) Contact angle measurement Analyses for evaluation of synthesized NPs and nanocomposites	Wettability alteration IFT reduction
14	Condensate blockage remediation in a gas reservoir through wettability alteration using natural CaCO_3_ nanoparticles (Ahmadi et al. 2019)	Calcium carbonate (bio-Ca)	Zeta potential measurements Contact angle measurements FESEM EDX Sand pack tests for different types of nanoparticles	Great wettability alteration
15	Silica nanofluid flooding for enhanced oil recovery in sandstone rocks (Youssif et al. 2018)	Silica	Core flood	Mobility control (by increasing the viscosity of injected fluid) Wettability alteration
16	Evaluation of Aluminium Oxide and Titanium Dioxide Nanoparticles for EOR Applications (Hogeweg et al. 2018)	Al_2_O_3_, TiO_2_	Viscosity measurement IFT measurement Micromodel injection	Mobility control IFT reduction
17	Synthesis of ZnO Nanoparticles for Oil–Water Interfacial Tension Reduction in Enhanced Oil Recovery (Soleimani et al. 2018)	ZnO	Adsorption test and analysis IFT measurement	Wettability alteration IFT reduction
18	Enhanced Oil Recovery of Low-Permeability Cores by SiO2 Nanofluid (Lu et al. 2017)	Silica	Contact angle measurement on quartz surface IFT measurement Core flood Viscosity measurement and evaluation of asphaltene content	Wettability alteration IFT reduction Asphaltene adsorption Viscosity reduction (oil)
19	Spontaneous Imbibition Investigation of Self-Dispersing Silica Nanofluids for Enhanced Oil Recovery in Low-Permeability Cores (Dai et al. 2017)	Surface-modified silica	FTIR DLS Zeta potential measurements Dynamic and static contact angle measurement IFT measurement Spontaneous imbibitions	Wettability alteration IFT reduction (abit)
20	The effect of nanoparticles on spontaneous imbibition of brine into initially oil-wet sandstones (Sobhani and Ghasemi Dehkordi 2019)	Silica	Spontaneous imbibition for different concentrations of nanoparticles	Wettability alteration
21	Evaluating the potential of surface-modified silica nanoparticles using internal olefin sulfonate for enhanced oil recovery (Ahmed et al. 2020)	Surface-modified silica, pure silica	Contact angle measurement IFT measurement Viscosity measurement Core flood	Wettability alteration IFT reduction Increasing viscosity of injected phase
22	Experimental investigation of the effect of green TiO2/Quartz nanocomposite on interfacial tension reduction, wettability alteration, and oil recovery improvement (Zargar et al. 2020)	SiO_2_/quartz (nanocomposite)	XRD FTIR SEM Contact angle measurement IFT measurement Core flood	Wettability alteration IFT reduction
23	Synthesis of silica nanoparticles with different morphologies and their effects on enhanced oil recovery (Khademolhosseini et al. 2020)	Synthesized silica (different morphologies)	SEM Contact angle measurement IFT measurement Micromodel injection	Wettability alteration IFT reduction
24	Microemulsions stabilized by in-situ synthesized nanoparticles for enhanced oil recovery (Hu et al. 2017b)	Synthesized iron oxide	IFT measurement Core flood Viscosity measurement Stability of microemulsions	Increase viscosity of injected fluid IFT reduction Stable microemulsions
25	Comparative study of different enhanced oil recovery scenarios by silica nanoparticles: An approach to time-dependent wettability alteration in carbonates (Keykhosravi et al. 2021)	Silica	Contact angle measurement IFT measurement Core flood	Wettability alteration due to shutoff IFT reduction
26	Hydrophilic Nanoparticle-Based Enhanced Oil Recovery: Microfluidic Investigations on Mechanisms (Xu et al. 2018)	Hydrophilic silica	Zeta potential measurement Micromodel injection (single pore scale and network of pores)	Swelling, dewetting and disjoining of oil (introduced by authors)
27	Novel smart water-based titania nanofluid for enhanced oil recovery (Shirazi et al. 2019)	TiO_2_ (at the presence of different ions)	Visual stability of nanoparticles Zeta potential measurement Particle size measurement Contact angle measurement IFT measurement Spontaneous imbibition	Wettability alteration (man mechanism) IFT reduction (negligible)
28	Impact of SnO2 nanoparticles on enhanced oil recovery from carbonate media (Jafarnezhad et al. 2017)	SnO_2_	Contact angle measurement IFT measurement SEM Core flood	Wettability alteration IFT reduction
29	Experimental Investigation of Aluminosilicate Nanoparticles for Enhanced Recovery of Waxy Crude Oil (Wijayanto et al. 2019)	Aluminosilicate	Observation of stability Amott index IFT measurement (spinning drop) Core flood	Wettability alteration IFT reduction
30	Silica Nanoparticles Suspension for Enhanced Oil Recovery: Stability Behavior and Flow Visualization (Li et al. 2018)	Silica	Contact angle measurement DLS Turbiscan stability index IFT measurement (spinning drop) Micromodel injection	Wettability alteration Emulsification
31	A novel nanofluid based on sulfonated graphene for enhanced oil recovery (Radnia et al. 2018)	Sulfonated grapheme	FTIR IFT measurement Contact angle measurement UV analyses Core flood	Wettability alteration IFT reduction
32	Enhanced waterflooding with NiO/SiO2 0-D Janus nanoparticles at low concentration (Giraldo et al. 2019)	NiO_2_/SiO_2_	TEM Zeta potential measurement IFT measurement (Wilhelmy plate) Contact angle measurement Viscosity measurement Core flood	IFT reduction (main mechanism) Increasing the viscosity of injected fluid Wettability alteration
33	Experimental study and numerical modeling for enhancing oil recovery from carbonate reservoirs by nanoparticle flooding (Sepehri et al. 2019)	Silica	Gas chromatography of oil Contact angle measurement Core flood	Wettability alteration
34	Application of Synthesized Silver Nanofluid for Reduction of Oil–Water Interfacial Tension (Khalilnejad et al. 2020)	Silver	DLS Zeta potential measurement IFT measurement	IFT reduction
35	EOR by Water Injection with Nanoparticles into a Carbonate Oil Reservoir (Akhmetgareev et al. 2019)	CaO, Silica, Al_2_O_3_	DLS Electrical conductivity and pH Rel. perm	IFT reduction Wettability alteration
36	Effect of a nanoparticle on wettability alteration and wettability retainment of carbonate reservoirs (Shi et al. 2022)	Surface-modified silica	DLS TEM Zeta potential measurement TGA Contact angle Imbibition test	Wettability retainment
37	Effect of surface functionalized silica nanoparticles on interfacial behavior: Wettability, interfacial tension and emulsification characteristics (Gholinezhad et al. 2022)	EOR-12 nanoparticles	SEM Zeta potential measurement Contact angle IFT measurement Contact angle Emulsification	Wettability alteration Emulsification
38	Improving the stability of nanofluids via surface-modified titanium dioxide nanoparticles for wettability alteration of oil-wet carbonate reservoirs (Hosseini et al. 2022)	Surface-modified TiO2 and unmodified TiO2	FTIR XRD SEM DLS test Zeta potential measurement Contact angle	Wettability alteration
39	Effect of 2D Alpha-Zirconium Phosphate Nanosheets in Interfacial Tension Reduction and Wettability Alteration: Implications for Enhanced Oil Recovery (Qing et al. 2022)	α-Zirconium phosphate	DLS SEM TEM IFT measurement Contact angle Core flood	Wettability alteration
40	The synergistic effect of Fe2O3/SiO2 nanoparticles concentration on rheology, wettability, and brine-oil interfacial tension (Hassan et al. 2022)	Fe2O3/SiO2	FTIR XRD FESEM EDX TGA IFT measurement Contact angle	IFT reduction Wettability alteration Stability enhancement Increasing the viscosity
41	Wettability alteration to maintain wellbore stability of shale formation using hydrophobic nanoparticles (H. Li et al. 2022a, b)	Hydrophobic silica	DLS SEM TEM FTIR TGA Uniaxial compressive strength tests Contact angle Imbibition test filtration test	Wettability alteration
42	Effects of modified graphene oxide (GO) nanofluid on wettability and IFT changes: Experimental study for EOR applications (Jafarbeigi et al. 2021)	Modified graphene oxide	FTIR XRD Zeta potential measurement FESEM viscosity measurement Adsorption experiments IFT measurement Contact angle Emulsification Core flood	IFT reduction Wettability alteration
43	Improving stability of iron oxide nanofluids for enhanced oil recovery: Exploiting wettability modifications in carbonaceous rocks (Toma et al. 2022)	Superparamagnetic iron oxide nanoparticles	FTIR DLS XPS TEM Imbibition test IFT measurement Contact angle	Wettability alteration
44	Preparation and characterization of modified amphiphilic nano-silica for enhanced oil recovery (Cao et al. 2022)	Modified amphiphilic silica	Element analysis FTIR TGA TDG TEM DLS Zeta potential measurement Emulsification IFT measurement Contact angle Micromodel test Core flood	IFT reduction Wettability alteration Emulsification
45	The effect of hydroxyapatite nanoparticles on wettability and brine-oil interfacial tension as enhance oil recovery mechanisms (Ngouangna et al. 2022)	Hydroxyapatite nanoparticles	FTIR EDX TEM Zeta potential measurement IFT measurement Contact angle Core flood	IFT reduction Wettability alteration
46	Performance evaluation and mechanism study of a functionalized silica nanofluid for enhanced oil recovery in carbonate reservoirs (Bai et al. 2022)	Functionalized silica	FTIR TEM SEM TGA NMR DLS Zeta potential measurement Viscosity measurement IFT measurement Contact angle Micromodel test Core flood	IFT reduction Wettability alteration Viscosity enhancement

### Functional groups of NPs

Some groups of atoms which are responsible for main characteristics and activities of a structure are known as functional groups (Bader et al. 1994). Each nanoparticle has unique functional groups which affect their performance and applications. Hydrophilicity or hydrophobicity, adsorption or desorption on the surface of rock and ability to reduce the IFT are some of the properties which could be determined by their functional groups (Salvador-Morales et al. 2009). Table 2 presents the functional groups and properties of some commonly used nanoparticles for EOR purposes.

**Table 2:** Functional groups and properties of some commonly used NPs for EOR purposes

Nanoparticle	Functional group	Properties
SiO_2_ (Montes et al. 2020)	Silanol (O–Si–H)	Acidic, strongly hydrophilic, forming strong hydrogen bonds with halide and acetate ions
Graphene (Chen 2019)	Hydroxyl	Polarity, forming strong hydrogen bonds, hydrophilic, amphoteric
	Carboxyl	Hydrophilicity, high melting and boiling point, forming hydrogen bonds
	Carbonyl	Polarity, high reactivity, attraction between molecules and high boiling and melting point
	Oxirane	Participating in addition reaction, water soluble
$\gamma$-Al_2_O_3_ (Amirsalari and Shayesteh 2015)	Oxy (Al-O)	Formation of strong hydrogen bond, high reactivity
	Hydroxy (Al–OH)	Solubility in water, hydrophilicity
TiO_2_ (Kumar et al. 2014)	Hydroxyl	Polarity, forming strong hydrogen bonds, hydrophilicity, amphoteric
	Carboxyl	Hydrophilicity, high melting and boiling point, forming hydrogen bonds
	Carbonyl	Polarity, high reactivity, attraction between molecules and high boiling and melting point
	Secondary Amine	Formation of hydrogen bonds

The existence of carbon included functional groups in some NPs relates to the synthesizing procedure. Stability of TiO_2_ NPs is a challenge for their application in oil reservoirs. The attraction force between carbonyl functional groups of TiO_2_ is responsible for agglomeration of these NPs (Kumar et al. 2014).

However, to change the properties of nanoparticles, they could be modified, functionalized or coated with different materials. Due to the properties and functional groups of coating materials, the properties of NPs will be changed. Functionalization of NPs and selection of coating materials depend on the condition of reservoirs (Al-Shatty 2022).

Wu et al. observed a great reduction in IFT of oil and water by functionalized silica NPs with 3-minopropyltriethoxysilane and lauric acid. This observation was because of amphiphilic structure of silica NPs and consequently their appropriate location at the interface of oil and water. Existence of aliphatic amines induces water solubility to the NPs (Wu et al. 2020). In contrast, hydrophobic alkyl parts reduce water solubility. Simultaneous existence of these functional groups makes the consisting materials hydrophobic (Nasr et al. 2021). In another study, Gholinezhad et al. used silica NPs functionalized by ethylene glycol functional groups to investigate wettability alteration. They observed the wettability of a SurfaSil-treated glass alters from oil wet toward water wet. Seating of carbon chains on the siloxane group (which is adsorbed to the surface of glass by SurfaSil) results a new surface with $\mathrm{OH}$ group of ethylene glycol and modifies the surface to water-wet condition (Gholinezhad et al. 2022).

Janus NPs are functionalized NPs which have two or more physical properties in surface. Surfactants are widely used for production of Janus NPs (Tohidi et al. 2022). Lou et al. modified graphene oxide NPs to attach alkyl chains on the surface of NPs. Graphene oxide NPs already have carbonyl and carboxyl functional groups. They reported 15.2% enhancement in oil recovery through core flooding experiments. In comparison with non-functionalized NPs, oil recovery was 3 times greater by application of functionalized NPs (Luo et al. 2016).

### Stability of nanofluids

Stability of nanofluids is dependent on various parameters such as existence of ions, size of nanoparticles, pH of fluid and temperature. Achievement of stable dispersions could be a destructive factor in water treatment process. The methods of stabilizing nanoparticles are subdivided into two main categories: physical and chemical methods (Wu et al. 2011). Any stabilizing process that suspend nanoparticles by application of mechanical force is considered as a physical method while chemical methods include the addition of some chemical agents like acid, surfactants, etc. (Wu et al. 2011).

Based on DLVO theory, attraction or repulsion between each pair of particles depends on electrical attractive and repulsive forces. Therefore, electrokinetic properties are of importance for stability of nanofluids (Dahirel and Jardat 2010). Electrokinetic properties could be governed by controlling pH of the media. Many of nanofluids are not stable in acidic pH ranges. But this could not be assumed as a general rule for all of the nanofluids. For example, graphite nanofluids reflect fair stability at pH values around 2 (Mukherjee and Paria 2013). Huang et al. achieved stability for dispersions contained Al_2_O_3_ and Cu nanoparticles at pH values between 8.5 and 10. They stabilized 1000 ppm of nanoparticles in deionized water using pH control method (Huang et al. 2009). Zhang et al. introduced procedure of synthesis and storage conditions as two effective factors for stabilization of nanofluids. After dispersion of nanoparticles in base phase, aggregates could be formed over time. They evaluated disaggregation of metal oxide nanoparticles by sonication and addition of HCl and MgCl_2_ to nanofluids. It was observed that synthesizing procedure may result in the formation of chemical bonds between nanoparticles and any effort would not disaggregate nanoparticles below a specified size. Also they stated that the storage of nanoparticles for more than 1 month can lead to aggregation (Zhang et al. 2008). In another study, addition of TiO_2_ NPs to SiO_2_ nanofluid (PAM + deionized water + SiO_2_) increased the stability of nanofluid. Addition of 0.05 wt% and 0.1 wt% of TiO_2_ NPs to the dispersions resulted in more 14 and 19 days of stability, respectively (Kumar and Sharma 2018).

Keller et al. examined the stability of TiO_2_, ZnO and CeO nanoparticles in various types of waters. To find out the appropriate condition for stability, they measured electrophoretic mobility of nanoparticles in each fluid. Electrophoretic mobility is defined as the velocity of suspended particles induced by an electrical field, divided by strength of electrical field. It is clear that the greater values of electrophoretic mobility show better stability. Equation 3 represents electrophoretic mobility.

3 $$\mu = \frac{v}{{E_{0} }}$$

where *μ* represents the electrophoretic mobility (m^2^/s), ${\varvec{v}}$ describes the velocity of particles (m/s) and *E*_0_ is the strength of electrical field (V/m). They observed that increasing the value of total organic carbon (which was considered as a representative for amount of organic compounds in water) leads to higher electrophoretic mobility and consequently more stability. In contrast to their predictions, they observed that increasing the ionic strength of water reduces the electrophoretic mobility and stability. They reported that the aggregation of all nanoparticles at low total organic carbon and high ionic strength was very high, but by increasing TOC^1^ and reducing the value of IS,^2^ the aggregation ceased. It was also observed that controlling the value of pH for achieving stability is an effective solution for deionized, distilled and neutralized water. But there is not a clear trend between pH and stability of nanoparticles in saline waters (Keller et al. 2010). According to importance of nanoparticles stability in water treatment process, many researchers investigated the stability of different nanoparticles in the presence of divalent ions and natural organic matters. As Zhang et al. expressed, adding low amounts of salts to nanofluids results in aggregation of nanoparticles while the existence of natural organic material induces a negative charge on the surface of particles and stabilizes the nanofluid. They observed a unique behavior for SiO_2_ nanoparticles. Due to low adsorption of natural organic material by SiO_2_ and small Hamaker constant, the presence of divalent ions and natural organic material does not affect the stability of SiO_2_ nanoparticles (Zhang et al. 2009).

Polymers also have the tendency to stabilize nanofluids by altering the surface. Surface modification of hydrogen-terminated silicon nanoparticles by an amphiphilic polymer resulted in a stable nanofluid (Zhang et al. 2007).

**Fig. 4:**
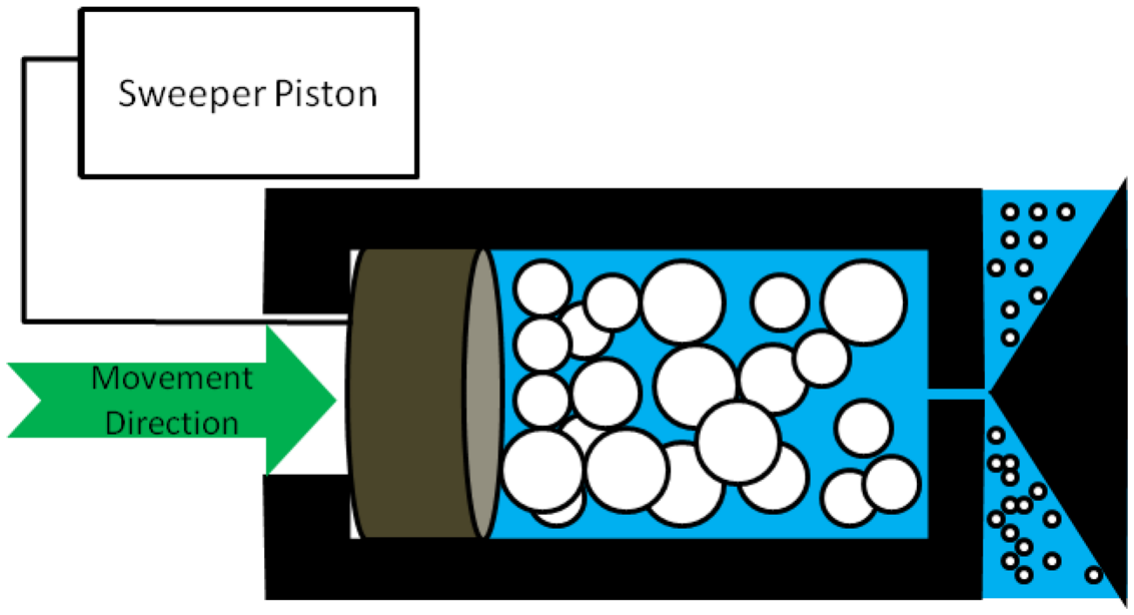
Operation of a high-pressure homogenizer

Hwang et al. tested different physical methods for stabilizing carbon black and silver nanoparticles. They examined the stability of nanofluids prepared by stirrer, ultrasonic disrupter, ultrasonic bath, high-pressure homogenizer and magnetron sputtering. The nanofluids which prepared by stirrer were mixed at 1500 rpm for 2 h. The ones which prepared by ultrasonic bath and ultrasonic disrupter were mixed with frequency at 40 kHz and 20 kHz, respectively. Both sonicating apparatuses have operated for 1 h. High-pressure homogenizer operated at 18000psi and each nanofluids passed 3 times through the system entirely. They reported that high-pressure homogenizer produces the most stable nanofluids among other physical methods. In this manner, ultrasonic disrupter, ultrasonic bath and stirrer were introduced as next effective devices, respectively. In addition, the nanofluids stabilized by magnetron sputtering method reflected the best stability among all methods. It could be concluded that chemical stabilizing methods are more effective than physical methods (Hwang et al. 2008). Figure 4 presents the performance of a high-pressure homogenizer schematically. It could be observed exerted pressure forces the nanofluid to leave the chamber through narrow tubes. The torque which is applied to the particles through this movement results in disaggregation of agglomerated nanoparticles (Anandharamakrishnan 2014).

One of the most effective parameters for stabilizing nanoparticles is pH. Aggregation of nanoparticles increases at zero-point charge. Therefore, pH should be considered during stabilization of nanofluids (Umh and Kim 2014)**.**

Tso et al. argued that the agglomeration of nanoparticles starts from the first moments of mixing. The aggregates could form up to microscale size. They used a stirrer at 15000 rpm to break the aggregates. The researchers observed that stirring can only break down the aggregates to micron sizes. To achieve a better disaggregation, ultrasonic instrument was tested. They found out ultrasonic is a more efficient way for breaking the aggregates. The broken aggregates were still much larger than the original size of nanoparticles. Also, they proved the existence of nanoorganic matter in water simplifies the procedure of stabilizing nanoparticles. Therefore, stabilizing nanoparticles in distilled and deionized water will be a harder task than in natural water (Tso et al. 2010). Keykhosravi and Simjoo investigated the effects of monovalent and divalent ions on stability by using NaCl and MgCl_2_. Measuring zeta potential, they found out that the presence of divalent ions in brine lowers the stability of silica nanoparticles and monovalent ions result in more stability in contrast. Results showed that more stability of silica nanoparticles is a positive effect to achieve more wettability alteration toward more water-wet state (Keykhosravi and Simjoo 2019). Xu et al. reached a stable nanofluid of iron oxide nanoparticles by using surfactant. They introduced coating process and surfactant-to-nanoparticle ratio as the main governing parameters for stability (Xu et al. 2011).

In another study, Abbood et al. stabilized CuO nanofluids by dodecyl-3-methylimidazolium chloride ([C12mim][Cl]) surfactant. Their nanofluids were stable for 1 month. Not only the stability condition improved, but also they observed the presence of 1000 ppm of surfactant boosted wettability alteration and synergistic effect of NPs and surfactant increases ultimate oil recovery up to 21.2% (Abbood and Hosseini 2022).

### Application of surfactant and nanoparticles

**Fig. 5:**
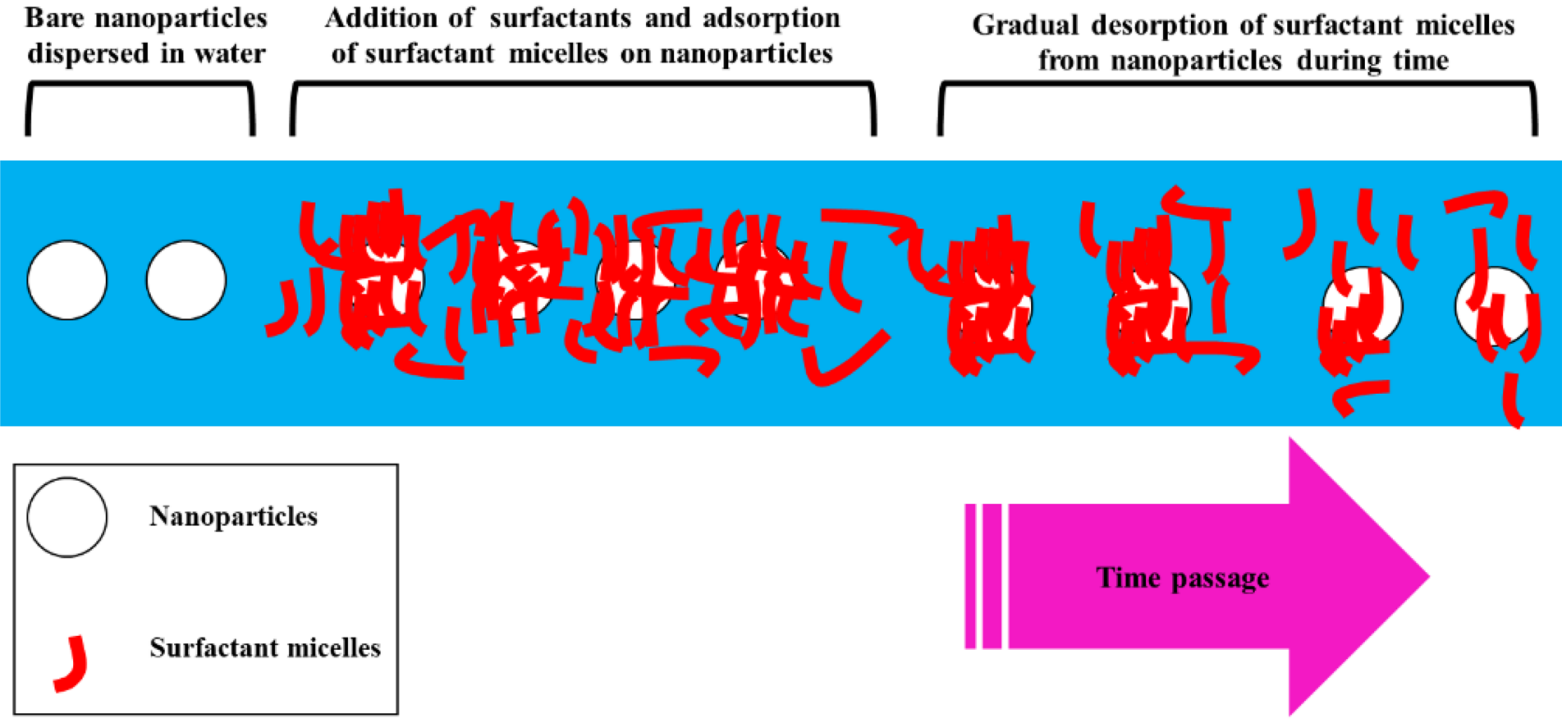
Schematic description of adsorption/desorption of surfactants by nanoparticles

Significant reduction in IFT is the main purpose of using surfactants. They have a low potential to alter the state of wettability (Golabi et al. 2009). In addition, some hybrid methods proved the addition of some divalent ions to surfactant solutions can empower wettability alteration mechanism (Hosseini et al. 2020). Application of nanoparticles with surfactant is known as a hybrid EOR method for achievement of more oil recovery. As it was aforementioned, surfactants are capable of increasing the stability of nanofluids. The more the stability of nanofluids, the more efficiency they might have. The main possible mechanism for stability is adsorption and desorption of surfactant by nanoparticles. Adsorption and gradual desorption of surfactants by nanoparticles could be a valuable point to improve the operation of surfactants (Olayiwola and Dejam 2019). Figure 5 illustrates the procedure of adsorption and desorption of surfactants by nanoparticles schematically.

Betancur et al. studied the adsorption of different surfactants on SiO_2_ nanoparticles. They also compared the performance of the mixture of nanoparticles and surfactant with surfactant alone. They observed that the critical micelle concentration (CMC) increases at higher temperature. They legitimated this phenomenon with disorganization of nonpolar groups in water molecules at high temperatures. Based on their reports, the adsorption of surfactant micelles on nanoparticles reduces at higher temperatures. This is due to exothermic nature of adsorption interaction. The authors designed two interesting procedures for preparation of nanoparticle and surfactant dispersion. At first procedure brine, surfactant and nanoparticles were mixed simultaneously. The second one included the addition of nanoparticles after preparing solution of surfactant and brine. They observed greater adsorption of surfactant on surface of nanoparticles using the latter one. The lower adsorption of surfactants during first procedure is related to a competition between surfactant molecules for either adsorption on nanoparticles or formation of micelles. This is a fair justification for lower size of formed micelles in the dispersions which was prepared by the first method. Finally, no impressive change on the reduction of IFT reported in the presence or absence of nanoparticles. However, recovery factor increased about 240% (comparing with application of surfactant solely) in the presence of SiO_2_ nanoparticles due to adsorption of micelles by NPs (Betancur et al. 2018).

Zhao et al. made an experimental research on potentials of nanofluids composed of deionized water, SiO_2_ nanoparticles and TX-100 surfactant for EOR applications. They compared the mechanisms of surfactant solutions and nanofluid-based surfactant solutions. They concluded that the addition of nanoparticles to the surfactant solutions does not change the value of IFT significantly. 16% increase in oil recovery during spontaneous imbibition tests by nanofluids is reported. This amount is twice of recovery achieved by surfactant solution. The dominant mechanism for the enhancement of oil recovery is attributed to higher wettability alteration. They checked the stability of nanofluids at various temperatures and salinities. Obtained results did not show any great change in stability by increasing temperature up to 70 °C (Zhao et al. 2018). Adsorption of surfactant on rock surface is known as a limitation parameter for efficiency of surfactant flooding (Belhaj et al. 2020). Nanoparticles could be used as an inhibitor for surfactant adsorption. Wu et al. evaluated static and dynamic adsorption of SDS surfactant on rock surface. The authors obtained dynamic adsorption by comparing the concentration of surfactant content between injected and effluent fluids. The results indicated a significant reduction in adsorption of surfactant on rock surface in the presence of nanoparticles. The ultimate recovery factor reported for injection of nanoparticle and surfactant dispersion is 7% greater than injection of surfactant solution solely (Wu et al. 2017). In another study, Abbood et al. investigated the addition of 1-dodecyl-3-methyl imidazolium chloride surfactant to SiO_2_ nanofluids. They pointed out NPs do not have significant effects on the reduction of IFT, but their synergistic effects with surfactant have great effects on wettability alteration. Finally, they found that application of NPs with surfactants results in production of extra 15.6% of synthetic oil (Abbood et al. 2022).

From all mentioned above, it could be concluded that although both NPs and surfactants are capable of reducing the IFT, but their mixture is not so effective in the reduction of IFT. Surfactants have the ability to stabilize NPs dispersions by inducing surface charge on NPs. Besides gradual desorption of surfactants by NPs can prevent retention of surfactants on surface of porous media and enhance the performance of surfactants. On the other hand, intensified wettability alteration could be considered as one of the main mechanisms for enhancing oil recovery by hybrid application of NPs and surfactant.

### Hybrid of polymer and nanoparticles

**Fig. 6:**
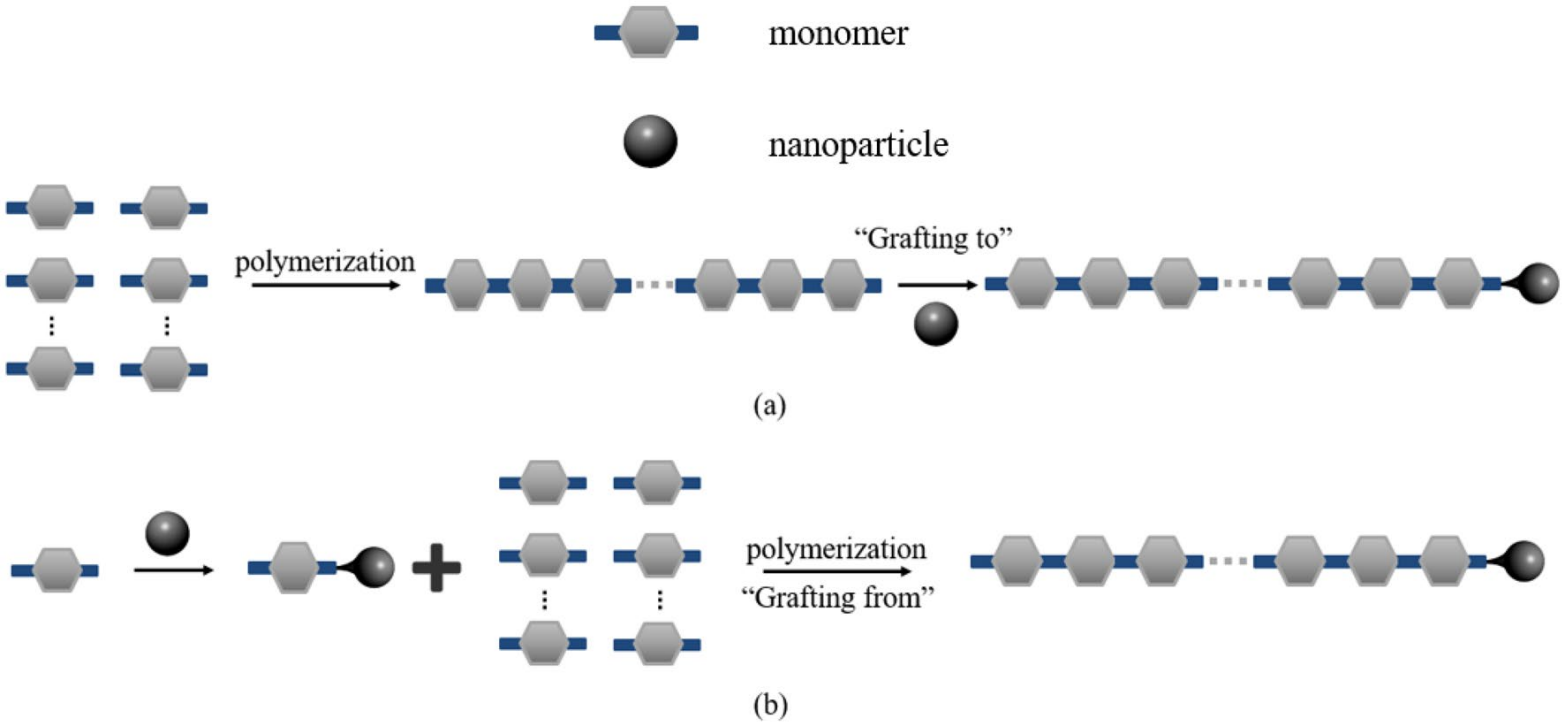
Schematic description of "grafting to" (**a**) and "grafting from" (**b**) methods

Polymer flooding is a promising EOR method which improves oil recovery mainly by mobility control. Mobility ratio is an important factor for governing macroscopic sweep efficiency. This method is used more than 50 years and it has proved that polymers can increase oil recovery up to 10–20% on average (Han and Lee 2014; Sheng et al. 2015). Like other EOR methods, polymer flooding has some limitations, e.g., viscosity loss due to shear rate and shear stress, retention in porous media and degradation under reservoir condition. Thus, the efficiency of the method is highly affected by reservoir conditions and fluid chemistry. To enhance the performance for application in harsh conditions researchers designed and investigated some NPs–polymer systems (Jan Bock Donald et al. 1987; Nourafkan et al. 2019; Tang et al. 2022; Ye et al. 2013; Zahiri et al. 2022). The synergistic effects of NPs and polymers reflected some promising results. In this manner, various types of NPs like silica (Hu et al. 2017a; Zeyghami et al. 2014; Zhu et al. 2014a, 2014b), titania (Cheraghian 2016), alumina (Cheraghian 2016; Minagawa and White 1976), iron (Kmetz et al. 2016; Tarek and El-Banbi 2015), zirconia, graphene and its derivatives (Haruna et al. 2019; Haruna and Wen 2019; Liu et al. 2012) and clay nanoparticles (Cheraghian 2015; Cheraghian et al. 2015; Cheraghian and Khalilinezhad 2015; Nezhad and Cheraghian 2016; Rezaei et al. 2016) are used. Combination of polymer and NPs could be done in two ways: (1) polymer grafted nanoparticles (PGN) and (2) hybrid of polymer nanofluid suspension (PNS). PGNs are chemical agents synthesized by attachment of polymer onto nanoparticle surface (Gbadamosi and Junin 2018). PGNs are created using two methods: “grafting to” and “grafting from.” Using “grafting to” method, the end-functionalized polymers react with an appropriate surface of NPs and “grafting from” method tries to grow polymer chains from an initiator-terminated self-assembled monolayer (Kango et al. 2013). Figure 6 shows the schematic description of PGNs synthesis using “grafting to” and “grafting from” methods. PNS is simply prepared by mixing or blending nanoparticles and polymer solutions (Gbadamosi and Junin 2018). In addition, sol–gel method could be used to synthesize polymer–NPs nanocomposites. Rezvani et al. synthesized chitosan @ Fe_3_O_4_ nanocomposites by this method. They mixed 0.5 ml of acetic acid with deionized water in a 50-ml volumetric flask. Then they added 0.125 g of chitosan powder to the mixture and stirred with a mechanical stirrer. In the next step, they added 1 g of Fe_3_O_4_ NPs to the mixture and stirred for 30 min. Finally, 25 ml of a solution contained deionized water and 1 g of NaOH added to the solution and stirred for 1 min. The solution filtered with paper and remained particles frozen at − 20 °C for 24 h (Rezvani et al. 2018b). Studies indicated that different NPs have different effects on polymer flooding performance and adding NPs to polymer solutions can improve chemical and thermal resistance, rheological behavior and also rock–fluid interactions (Cheraghian et al. 2014; Khalilinezhad et al. 2017; Li et al. 2010; Paul and Robeson 2008; Pavlidou and Papaspyrides 2008).

Saha et al. studied the synergistic effects of silica–xanthan composite on enhancing oil recovery from sandstone cores at low (30 °C) and high (90 °C) temperatures. They reported that silica NP-assisted polymer flooding enhances oil recovery about 20.82% and 18.44% at 30 °C and 90 °C, respectively. Wettability alteration, IFT reduction, higher viscosity and more stable emulsions were responsible for enhancing the amount of recovered oil. They also observed that in contrast to formation water, silica NPs were stable in the polymer solutions (Characteristics et al. 2018). Alaminia and Khalilinezhad investigated the effects of hydrophilic silica nanoparticles and their size on  polyacrylamide (PAM) solutions. They reported using silica with PAM increases the viscosity of polymer solution. Besides, larger size of silica NPs reflected greater efficiency in this manner (AlamiNia and Khalilinezhad 2017). Khalilinezhad et al. used experimental tests and numerical simulation to examine the effects of silica and clay on flow behavior of polymer solutions. The results showed that using silica and clay not only increases the viscosity, but also reduces the retention of polymer in porous media. Clay reflected less efficiency on adsorption and viscosity in comparison with silica (Khalilinezhad et al. 2017, 2016).

Rellegadla et al. studied the effects of adding nickel NPs to xanthan gum solution on oil recovery. They observed that NPs can increase the intrinsic viscosity of polymer solution and also enhance oil recovery compared with application of NPs and polymer individually. The achieved additional oil recovery by using xanthan gum solution and nickel NPs is equal to 5.98%. The additional oil recovery obtained by using xanthan solution and nickel NPs dispersion individually was 4.48% and 4.58%, respectively (Rellegadla et al. 2018). Khan et al. studied the rheological behavior of different mixtures of polymer and SiO_2_, TiO_2_ and Fe_2_O_3_ NPs at 50 °C, separately. Different concentrations of NPs in the range of 0–1 wt% were used with 1 wt% of HPAM. The results showed that the highest concentration of each NPs has the most effect on increasing viscosity. SiO_2_, TiO_2_ and Fe_2_O_3_ enhanced the viscosity of polymer solution (at shear rate of 100 1/s) from 0.002 cp to 0.005 cp, 0.3 cp and 0.016 cp, respectively. The authors claimed using NPs increases storage module of polymer solutions. NPs–polymer core flooding was also performed and comparing to conventional polymer flooding improved recovery is reported (Khan et al. 2018). Corredor et al. synthesized polyacrylamide-grafted SiO_2_, TiO_2_ and Al_2_O_3_. Their analysis proved NPs grafted polymers enhance the viscosity, lower the IFT and alter the wettability in the presence of NaCl at 25 °C. (Corredor et al. 2019a). They also investigated the rheological behavior of mixtures of xanthan and SiO_2_, TiO_2_, Al_2_O_3_ and Fe(OH)_3_ NPs at 25 °C and different salinities. They concluded that the addition of TiO_2_, Al_2_O_3_ and Fe(OH)_3_ reduces the viscosity of xanthan solution. Contrarily, SiO_2_ enhances the viscosity of polymer solution (Corredor et al. 2018, 2019b).

Maghzi et al. conducted a series of experiments to investigate the effects of silica NPs on performance of polymer (PAM) solution for enhancing oil recovery. They examined the rheological behavior of polymer solution at various shear rates (0.001–3.486 1/s). It was concluded that adding silica NPs results in higher viscosity of solution. By flooding in micromodel, they observed 25% more oil recovery for NPs–polymer solution (Maghzi et al. 2013). They also assessed the synergistic effects of silica NPs and HPAM on wettability alteration of a glass micromodel. The authors found out the dispersions alter the wettability of micromodel toward strongly water-wet state (Maghzi et al. 2011).

Haruna et al. evaluated the potentials of using SiO_2_ and modified SiO_2_ with PAM to enhance oil recovery. They stated that using SiO_2_-PAM mixture have some limitations like agglomeration in harsh conditions. Chemical agent (3-aminopropyl) triethoxysilane was used to modify the surface of SiO_2_ for optimization of the interactions between functional groups of PAM and SiO_2_ in order to improve dispersion stability. The surface-modified SiO_2_ (M_SiO_2_) interacts with PAM and creates a protective shield on PAM micelles. So, they are capable of stabilizing the solution. Thermal stability also increased by using M_SiO_2_. Viscosity loss of M_SiO_2_-PAM solution after 70 days was just 10% while for SiO_2_-PAM and PAM system it was about 45% and 78%, respectively (Haruna et al. 2020).

Using ZrO_2_ NPs with polymer (PAM) solution at different temperatures and salinities has also studied by Al-Anssari et al. They studied the stability and viscosity of NPs–polymer systems. They have claimed that using zirconia NPs in small quantities (< 0.03 wt%) could improve solutions viscosity at high temperatures and high salinities. It is noteworthy that the adsorption of the NPs on polymer micelles occurred at low concentrations and the addition of extra amounts of zirconia NPs makes no significant effect (Al-Anssari et al. 2021). Table 3 summarizes some studies on hybrid application of NPs–polymer.

**Table 3:** Some studies of synergistic combination of NP and polymer

No	Researcher	Year	Polymer (Conc.)	NP (Conc.)	Temperature (°C)	Porous media	Porous media property	Salinity	Mechanism or observations
1	Hu et al. (2017a)	2017	HPAM (1000–10000 PPM)	SiO_2_ (1 wt%)	25 & 85	–	–	80,000 ppm	Solution viscosity improved significantly in high salinity and temperature
2	Khalilinezhad et al. (2017)	2017	HPAM (0.125 wt%)	SiO_2_ (0.45 wt%)	55	Sandstone	*K* = 5.6 mD, *φ* = 21%	28,000 ppm	Reduction of mobility ratio and polymer adsorption
3	Saha et al. (2018)	2018	Xanthan (5000 ppm)	SiO_2_ (0.1–0.5 wt%)	25 & 75	Berea sandstone	*K* = 700–1000 mD, *φ* = 25%	3000–30,000 ppm	No significant effect on polymer viscosity
4	Abdullahi et al. (2019)	2019	HPAM (2000 PPM)	Al_2_O_3_, SiO_2_, TiO_2_ (0.1 wt%)	0	Sand pack	*K* = 4 D, *φ* = 30%	3000 ppm	More stable solution against salinity and temperature and viscosity improvement
5	Haruna et al. (2019)	2019	HPAM (0.005–0.05 wt%)	GO (0.01–0.1 wt%)	25 & 85	–	–	–	Thermal stability and rheological behavior improvement in high salinity
6	Gbadamosi et al. (2019a)	2019	HPAM (500–5000 ppm)	SiO_2_ (0–1 wt%), Al_2_O_3_ (0–1 wt%	27–90	Sandstone outcrop	*K* = 168 md, *φ* = 15%	0.5–3.41 wt% NaCl	Rheology behavior improvement and wettability alteration to more water-wet condition, NP caused less polymer degradation
7	Kumar et al. (2020)	2020	HPAM (0.5 wt%)	CuO (0.02–0.1 wt%) + Nanoclay (0.04–0.12 wt%)	85	Sandstone	*K* = 5 D, *φ* = 33.5 T	10,000 ppm KCl	Using 2 kinds of NP increased polymer solution viscosity, improved viscoelastic properties and decreased polymer chemical and mechanical degradation
8	Aliabadian et al. (2020)	2020	HPAM (500 ppm)	S-GO (0.2–0.5 wt%) & E-GO (0.2–0.5 wt%)	24	Sand pack	*K* = 5–6.5 mD, *φ* = 35–40%	–	Viscosity and viscoelastic property decreased
9	Agi et al. (2020)	2020	HPAM (0.1 wt%)	SiO_2_ (0.1 wt%), Al_2_O_3_ (0.1 wt%), CSNP (0.2 wt%)	–	Sandstone	*K* = 201 mD, *φ* = 17%	0.9–2.2 wt%	IFT reduction and wettability alteration
10	Elhaei et al. (2021)	2021	HPAM (0.2 wt%)	SiO_2_ (0.05 & 0.1 wt%)	–	Sandstone	*K* = 10–12 mD, *φ* = 18.6–19.3%	–	Rheological behavior improvement
11	Santamaria et al. (2021)	2021	HPAM (0.05 wt%)	SiO_2_ (0–3000 ppm)	–	Micromodel	*K* = 5.7 D, *φ* = 70%,	–	No significant change in IFT, wettability alteration to more water-wet condition, the existence of NP improved viscoelastic behavior of NP–polymer solution and decreased the size of oil clusters
						Sand pack	*K* = 512–612 mD, *φ* = 21–26%		
						Sand outcrop	*K* = 72–123 mD, *φ* = 11–16%		
12	Khalilinezhad et al. (2021)	2021	HPAM (0.1–0.4 wt%)	Hydrophilic Silica Nanoparticle (0.1 wt%)	50	Carbonate stone	*K* = 20 mD, *φ* = 18%	0.8–1.4 wt.%	Using hydrophilic NP effects more on low molecular polymer which significantly is affected by polymer concentration, controlling mechanism is suggested to be mobility improvement and wettability alteration
						micromodel	*φ* = 0.39%		

### Hybrid of low-salinity water and nanoparticles

Addition of nanoparticles to low-salinity phase is an interesting topic for researchers. Numerous criteria including existence of ions, compatibility of NPs with composition of water and appropriate concentration of NPs should be considered for simultaneous application of nanoparticles and low-salinity phase. Existence of ions in bulk phase of nanofluids affects the stability of nanoparticles strongly. There are some methods recommended for stabilization of NPs in various ranges of salinity. Jafari et al. stabilized hydrophilic silica in seawater by using H^+^ protection. This method refers to add some amounts of HCl to nanofluid. The generated H^+^ ions protect the NPs from free ions in the bulk and increase the stability of nanofluids (Sofla et al. 2018). Addition of surfactant to the nanofluids is another method to stabilize NPs in saline solutions. Surface modification of NPs caused by adsorption of surfactant enhances the stability of NPs, especially in saline solutions (Olayiwola and Dejam 2019).

Wettability alteration is known as the main EOR mechanism of both low-salinity flooding (Hosseini et al. 2015) and nanoparticles injection. Numerous studies examined the application of various nanoparticles with low-salinity phase for different intentions. Taleb et al. investigated the optimum conditions for injection of low-salinity phase and nanofluid (composed of their synthesized Faujasitr-Based silica NPs) by static analyses. The low-salinity phase of their study was composed of 2 wt% NaCl and 0.2 wt% KCl. It was observed that increasing the concentration of synthesized NPs (up to 200 ppm) reduces the value of IFT. Contact angle measurements illustrated that the use of low-salinity phase containing nanoparticles makes the surface of the rock more water wet. Finally core flood tests showed 5% greater oil recovery by injection of low-salinity phase solely and 10% higher recovery factor by application of low-salinity-based nanofluids (Taleb et al. 2020). In another study, Sadatshojaei et al. evaluated the synergistic effects of using nanoparticles and low-salinity phase in a carbonate rock. Low-salinity phase (dilutions of seawater with TDS^3^ of 47,681.3 ppm) was composed of Na^+^, K^+^, Mg^2+^, Ca^2+^, SO_4_^2−^, Cl^−^ and HCO_3_^−^ ions. They categorized the existed ions into active and inactive ions. As they reported category of inactive ions includes Na^+^, K^+^ and Cl^−^ and active ions category consists of Mg^2+^, Ca^2+^, SO_4_^2−^. IFT and contact angle measurements proved that at lower concentrations of inactive ions, the actives would be capable of moving freely through the bulk phase and decrease the value of IFT. Also they concluded that increasing the salinity makes the nanofluid instable (Sadatshojaei et al. 2019).

Shakiba et al. added some amounts of silica nanoparticles to low-salinity water to stabilize instable sands during production from unconsolidated rocks. Since sands could be mobilized by injection of low-salinity water, precipitation of silica nanoparticles stabilizes unconsolidated sands. They reported that flooding the cores by low salinity and silica NPs enhances the strength of rock up to 46% more than the rocks which flooded by low-salinity phase solely (Shakiba et al. 2020).

To seek EOR potentials of low-salinity NPs system in heavy oil reservoirs, Ding et al. evaluated the performance of Al_2_O_3_ and SiO_2_ nanoparticles when dispersed in low-salinity phase. They selected a 1/10 dilution of brine containing Na^+^, Ca^2+^, Mg^2+^, Cl^−^, OH^−^, HCO^3−^, CO_3_^2−^ and SO_4_^2−^ as the low-salinity phase for their study. The addition of SiO_2_ nanoparticles to low-salinity phase at low temperature (25 °C) had no effect on oil recovery (before and after breakthrough), while injection of SiO_2_ nanofluid after low-salinity phase (as the second slug after low-salinity water injection) showed more than 2% increase in oil recovery. Despite SiO_2_, addition of Al_2_O_3_ NPs to the low-salinity phase resulted in much better sweep efficiency before breakthrough. But they realized that the amount of enhanced oil recovery after breakthrough of low-salinity containing Al_2_O_3_ nanoparticles is the same as what they observed for SiO_2_ nanoparticles. Since heavy oil is used in this study, increasing the temperature of injected phase resulted in higher recovery factor. At temperature of 45 °C, the low-salinity phase contained SiO_2_ showed a greater recovery factor than the one composed of Al_2_O_3_ nanoparticles. This trend is reported to change inversely at 70 °C (Ding et al. 2019).

By dispersing different concentrations of silica nanoparticles into dilutions of Persian Gulf seawater, Saeedi Dehghani and Daneshfar investigated the synergistic contribution for application of silica nanoparticles and low-salinity phase. They measured contact angle and performed some micromodel analyses in the presence of synthetic oil. They found out injection of silica nanoparticles dispersed in deionized water has lower efficiency than injection of low-salinity phase alone. Also, they observed a synergistic effect for injection of dispersed nanoparticles in the low-salinity phase. Since the addition of nanoparticles increased the viscosity of injected phase, better mobility control could be obtained using this method. The improved mobility control is capable of postponing breakthrough time (Dehaghani and Daneshfar 2019).

**Fig. 7:**
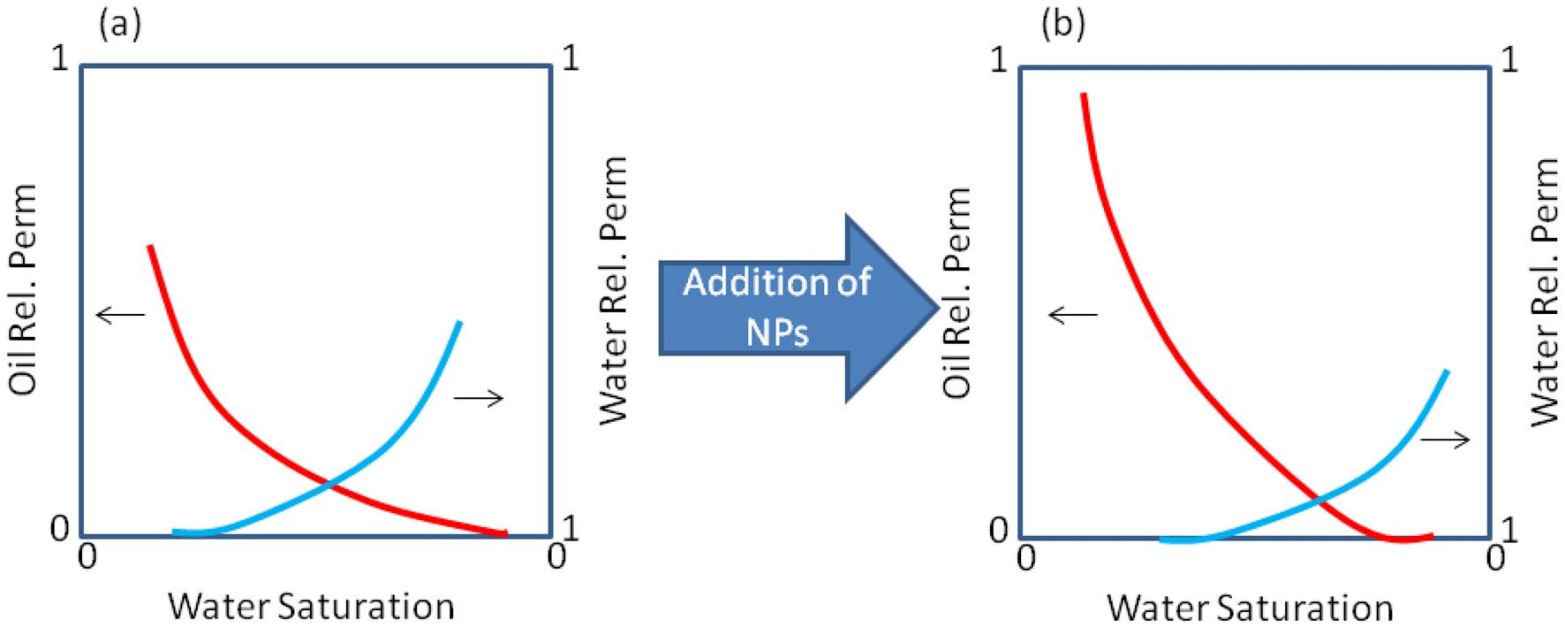
**a** Relative permeability curves in an oil-wet rock **b** and after the addition of NPs which results in wettability alteration

In another study, Sagala et al. functionalized silica nanoparticles and evaluated the capability of increasing oil recovery by injection of nanofluid-based low-salinity water. Their chosen low-salinity phase composed of 0.1 wt% of NaCl. Application of low-salinity water with surface-modified nanoparticles caused wettability alteration in oil-wet sandstones. Addition of nanoparticles to low-salinity phase also increased the value of recovery factor by 15% in comparison with injection of low-salinity phase alone. Their report also indicated a right shift of relative permeability curve after injection of low-salinity phase, while the movements of curves are greater in the presence of nanoparticles, which shows intensified wettability alteration (Sagala et al. 2020). The shift of relative permeability curve after injection of low-salinity phase and low-salinity-based nanofluids is illustrated schematically in Fig. 7.

Abhishek et al. investigated the adsorption of silica nanoparticles to the calcite and chalk surfaces under static and dynamic conditions and at different ranges of salinities. They measured the amounts of calcite and magnesium contents at inlet and effluent phases during core flooding by low-salinity phase included nanoparticles. They observed a reduction in calcite content in the effluent after addition of 0.1 wt% nanoparticles to the low-salinity phase. This could be a good sign to conclude calcite dissolution during low-salinity phase injection will be avoided by the addition of nanoparticles (Abhishek et al. 2018).

Kiani et al. examined using Al_2_O_3_ nanoparticles for injection into sandstone reservoirs at various salinity and temperature conditions. They obtained the most recovery factor at elevated temperatures up to 80 °C. Clay could be detached from the surface of sandstone and wettability alteration can take place during injection of low-salinity phase. High temperature is a positive factors for more adsorption of Al_2_O_3_ on the surface of rocks. Therefore, due to the occurrence of wettability alteration and stabilization of clay particles, the most recovery factor is reported at highest temperature (Kiani et al. 2016).

Divandari et al. analyzed the effects of salt type (NaCl, MgCl_2_ and CaCl_2_) on IFT in the presence of 3 different nanoparticles (Fe_3_O_4_, Fe_2_O_3_, SiO_2_). Nanoparticles were coated by citric acid. They introduced MgCl_2_ as the best IFT reducer among others when saline water is injected. This could be justified with respect to lower radius of Mg^2+^ in comparison with other ions. The shorter the radius of ions, the more effectiveness in the reduction in the IFT will take place. They reported that the minimum IFT values for all salts belongs to the concentration of 40000 ppm. Higher concentrations of salts resulted in accumulation of cations at the interface and restrict the tendency of asphaltenes for move toward interface. They introduced Fe_3_O_4_ as the most efficient nanoparticles for the reduction in the IFT. Also, they reported Fe_2_O_3_ as the less effective nanoparticle for the reduction in the IFT. The trend of IFT reduction (the most reduction was for MgCl_2_, CaCl_2_ and NaCl, respectively) was not changed by the addition of NPs or surfactant (Divandari et al. 2020). In fact, asphaltenes and resins are natural surfactants in crude oil. The addition of salts and nanoparticles can enhance or restrict their performance in the reduction in the IFT (Pejmannia et al. 2022).

Rezvani et al. made an extensive study on stability and efficiency of Al_2_O_3_ nanoparticles at porous media conditions. They measured the values of IFT and interfacial shear viscosity^4^ between synthetic oil (composed of toluene, *n*-heptane and asphaltene) and nanofluid at different temperature conditions and in the presence of MgSO_4_ and NaCl salts. Due to their results, increasing temperature decreases IFT. They reported that the rate of IFT reduction empowered in the presence of nanoparticles at some concentrations (Rezvani et al. 2019). Increasing temperature activates two mechanisms for decreasing IFT: (1) displacement of nanoparticles to the interface of oil and water and consequently increasing the surface (Ngai and Bon 2014), and (2) catalytic behavior of nanoparticles at elevated temperatures for cracking the heavier molecules of oil.

There are several points which should be considered for application of nanoparticles and low-salinity phase. Due to what mentioned above, researchers and operators should consider the following parameters:

1. Low-salinity phase decreases the IFT between oil and water with respect to repulsion of charges.
2. Density of ions charge plays a key role for activation of EOR mechanisms.
3. Increasing temperatures intensifies Brownian motion of nanoparticles. This is the reason for more efficiency of nanoparticles at elevated temperatures.
4. Active ions are composed of divalent ions and inactive ions include monovalent ions. Active ions are effective for the reduction in the IFT. Also, the performance of active ions improves at lower concentrations of inactive ions.
5. Instability of nanofluid accelerates in the presence of active ions. Stability of nanoparticles decreases at low concentrations of Mg^2+^.
6. There is an optimum concentration for nanoparticles to prevent formation of scale.
7. Sand production could be avoided by injection of some amount of nanoparticle with low-salinity water.
8. Better sweep efficiency is expected with addition of nanoparticles to the injected low-salinity water.
9. There is a synergistic effect for application of nanoparticles with low-salinity water. This effect empowers the mechanisms of each agent.

### Hybrid of nanoparticles and foam

Gas injection is faced with challenges like channeling, gas override, low sweep efficiency, fingering and unfavorable mobility ratio (Andrianov et al. 2012; Yang et al. 2019). About 70 years ago, foam injection became popular as a method that eliminates most of the aforementioned challenges (Sun et al. 2014) and now is a common EOR method (Hu et al. 2020; Jin et al. 2020; Zhou et al. 2020). Due to higher viscosity, it is also reported that the foam could have a viscosity up to 1000 times greater than gas (Liu et al. 2005). Observations showed that using foam, can be useful in heterogeneous porous media and divert the fluid to un-swept zones (Blaker et al. 2002; Hou et al. 2018; Skauge et al. 2002; Sun et al. 2019). Foam in porous media is defined as a gas dispersion within the liquid phase where continuous phase is a liquid and the discontinuous phase is a gas. The phases are separated by lamella (the thin film of liquid) (Almajid and Kovscek 2016; AlYousef et al. 2020; Falls et al. 1988). Stability is a key parameter which must be considered for application of foams (Bai et al. 2010; Guo and Aryana 2016; Ibrahim et al. 2017; Risal et al. 2019; Yang et al. 2017). Some factors like reservoir condition (e.g., reservoir temperature, pressure, oil saturation and composition, brine saturation and composition), foaming agent and its concentration and type of gas affect foam stability(Almubarak et al. 2020; Grigg et al. 2004). Thus, for EOR purposes, the longer the lifetime of the lamella, the greater the stability of the foam will be achieved (Zhu et al. 2004). Some surfactants and polymers could be used as foam stabilizers (Yekeen et al. 2018). Due to sensitivity of surfactants and polymers to high salinity and temperature (Babamahmoudi and Riahi 2018; Farzaneh and Sohrabi 2015; Ko and Huh 2019; Kutay and Schramm 2004; Lee et al. 2015; Singh and Mohanty 2017; Yekeen et al. 2017), recently novel methods such as combination of foams and nanoparticles (Almubarak et al. 2020) have been suggested as a solution to improve the stability of foams during flooding. Studies have shown that the use of nanoparticles as foam stabilizer leads to beneficial effects (X. Li et al. 2022a, b). The effects of TiO_2_ on foam stability and efficiency of oil production in glass micromodel were examined by Panahpoori et al. They observed that the mixture of TiO_2_ and hexadecyltrimethylammonium bromide (CTAB) improved foam stability. Results showed that adsorption of CTAB molecules on the surface of TiO_2_ NPs is the main reason for improvement in the stabilization of foam. They reported the most adsorption belongs to 0.03 wt% of CTAB and 0.03 wt% of TiO_2_ NPs. Also, micromodel flooding tests showed that nano-CTAB foam resulted more sweep efficiency (54%) and recovery factor than nano-CTAB flooding (Panahpoori et al. 2019).

In order to design a suitable foaming agent, Kumar et al. used carbon dioxide gas, Sodium dodecyl sulfate as anionic, CTAB as cationic and polysorbate 80 (Tween 80) as nonionic surfactants, silica, alumina, zirconium oxide, calcium carbonate and boron nitride nanoparticles and polymer, alcohol and alkali as additives. They observed that ionic surfactant can result in more stable foam in comparison with nonionic surfactant. Also adding nanoparticles improved foam stability. Specially using boron nitride reflected the best response among other nanoparticles (Kumar and Mandal 2017).

Almubarak et al. evaluated the role of nanoparticles on stabilization of foam. They combined a cationic surfactant and a surface-modified silica nanoparticle and conducted some glass micromodel tests to measure foam stability. They observed that using nanoparticle with surfactant decreases the mobility, improves sweep efficiency and enhances foam stability due to forming smaller bubbles (Almubarak et al. 2020).

Harati et al. investigated the effects of different gas types including nitrogen, methane and carbon dioxide on foams which stabilized by SiO_2_ nanoparticles and SDS. Results showed that the half time and oil recovery of methane, nitrogen and carbon dioxide foams at optimum nanoparticle concentrations are 1054 min with 25% R.F, 1720 min with 31% R.F and 62 min with 19% R.F, respectively (Harati et al. 2020).

The synergistic effects of alpha olefin sulfonate (AOS^5^) and molybdenum disulfide (MoS_2_^6^) nanosheets on foamability and recovery improvement are assessed by Raj et al. Their results illustrated that the synergy of AOS-MoS_2_ improves foam stability in the presence of calcium and sodium ions because the MoS_2_ nanosheets forms a layer around the lamella and protects it. They also reported that flooding by foams including AOS-MoS_2_ increases oil recovery by 12.1% in comparison with surfactant flooding alone (Raj et al. 2020).

Sakthivel and Kanj studied the effects of adding carbon nanodots to surfactant in order to enhance foam stability. They reported using carbon nanodots can improve foam stability in high-salinity condition (up to 70%) by increasing the lamella thickness and also can cause improvements in mobility control. Moreover, static tests showed that air and nitrogen foams are more stable than carbon dioxide (Sakthivel and Kanj 2021).

To discuss the effects of nanoparticle on foam system, Li et al. investigated the effects of nanoparticles on foam performance and wettability of carbonate rock. They observed that by increasing Silica nanoparticle concentration, foaming volume^7^ decreases while the generated foam is more stable. They also reported that increasing the concentration of nanoparticle alteres the state of wettability to more water-wet. Secondary surfactant foam and nanoparticle–foam flooding tests were conducted after water flooding and resulted in 28.6% and 37.5% oil recovery improvement (Li et al. 2020).

Liu et al. studied the effects of hydrophobicity of nanoparticles in nanoparticle–foam system. They used Fe_3_O_4_ with four different contact angles (12.7°, 20.6°, 57.5° and 94.3°). Results showed that nanoparticle modification can affect foam stability where the foam included nanoparticle with contact angle of 94.3° were 2.36 times more stable than non-modified one. Also a higher oil recovery than others achieved for mentioned nanoparticle–foam system (Liu et al. 2020). Zhao et al. synthetized and used amphiphilic surface-modified silica nanoparticles to improve foam stability and oil recovery. Their results demonstrated that the half-life of modified silica foam increased about 5 min at 60 °C in comparison with unmodified silica foam and flooding test also showed that modified silica foam system can increase oil recovery factor by 19.8% (Zhao et al. 2021).

Considering recent studies, between various nanoparticle types, silica is the most used nanoparticle for foam stabilization (Yekeen et al. 2018). Also, nanoparticles not only improve the foam stability, but also enhance foam performance in porous media by diverting injection foam to low-permeability zones and improving sweep efficiency.

### Advantages, disadvantages and limitations

**Fig. 8:**
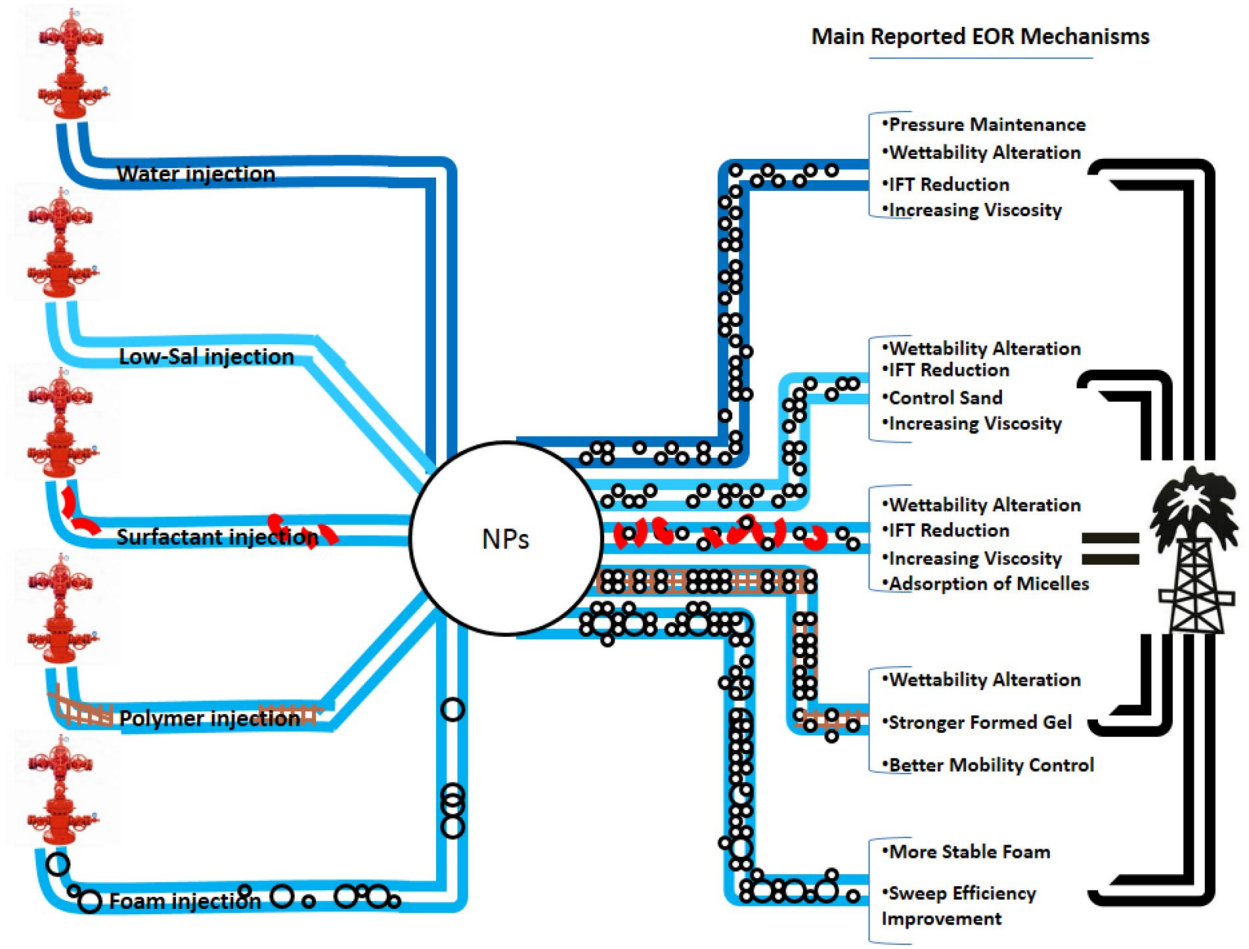
Schematic illustration of the main advantages of using NPs in EOR procedures

As discussed before applications of nanoparticles for EOR intends have great potentials. On the other hand, the synergy of using NPs with cEOR methods improves the performance of dominant contributing mechanisms. Figure 8 illustrates some main advantages of using NPs in EOR procedures based on the results of the literature reviewed above.

The usage of NPs in EOR process could not be considered as a complete, secure and perfect way. Compared to the other common EOR methods like water flooding, gas flooding and polymer flooding, hybrid nanoparticle EOR methods are too young and immature as they are only used in field scale in few and limited projects. Therefore, it is needed to investigate and study hybrid nanoparticle EOR methods comprehensively from different aspects to find the optimum way of using them. Based on several studies, Table 4 presents some of the main limitations and disadvantages for application of NPs in EOR procedures (Agista et al. 2018; Corredor et al. 2019c; Davoodi et al. 2022; Gbadamosi et al. 2019c; Kumar et al. 2022).

**Table 4:** Disadvantage/limitations of using hybrid of NPs and EOR methods

Disadvantage/Limitation	Description
Immaturity	Considering the lifespan of hybrid nanoparticle EOR methods in comparison with common EOR methods like water flooding, gas flooding and polymer flooding, they could be classified as immature method
Large-scale uncertainties	Using hybrid nanoparticle EOR methods in field scale has rarely been used and have been studied mostly in laboratory scale
Performance uncertainty in reservoir conditions	Salinity and temperature are two very effective and important factor in screening EOR methods. Unlike silicate nanoparticle and considering numerous nanoparticles used in petroleum industry, there are still limited investigations that study these important factors on the method performance. Also, most of the current articles which have studied the effect of harsh condition still have not capture the real status of reservoir condition in terms of salinity, hardness, ionic compounds, temperature and pressure
Economic studies and econometrics	In last recent years numerous nanoparticles have been proposed for enhancing oil recovery which have shown very good performance. But still a very important point has been missed out: economic aspects. Many synthetized nanoparticles have been produced and used on laboratory scale and have not reached mass production yet and there are open questions about the profit and expenses of their usage which should be noticed
Environmental effects	Most of the petroleum engineering studies related to nanoparticles have dealt with oil recovery, contributing mechanisms in enhancing oil recovery and rarely economic studies. Also, in last two decades legislation of environmental issues has been accelerated. Therefore, considering numerous types of nanoparticles used, the environmental effects of nanoparticles should be investigated and modeled in larger scale
Amount of nanoparticles used	In most studies using hybrid EOR methods of nanoparticles the amount of nanoparticle to base components is too high (twice to ten times higher) which raises the question that whether the performance of the basic component has been improved by nanoparticles or vice versa

Considering aforementioned advantages, disadvantages and limitations, the following suggestions could be taken into account for future related studies:

- Some important factors like reservoir condition, the main contributing mechanism and rock and fluid interactions are not fundamentally investigated.
- Environmental issues should be considered as one of the screening criteria factors for application of nanoparticles in EOR methods. Therefore, studies on the environmental effects of various nanoparticles used in EOR process would be interesting and helpful to select the best nanoparticle.
- Simulation and modeling studies make a great view of the performance of EOR methods and there is still lack of appropriate simulation and modeling studies, especially for large-scale application of NPs.
- Shape, size and aspect ratio are important intrinsic properties of nanoparticles which should be studied and tested comprehensively. The number of existing studies is not sufficient and no certain conclusion could be derived on the obtained results.
- Functional groups of nanoparticles determine their usage and play a key role in their performance. Therefore, investigating the type and variety of functional groups of nanoparticles, especially newer ones (like carbon-based nanoparticles), seems necessary.

### Economic evaluation of EOR process

It is forecasted that COVID-19 pandemic would have a great effect on energy consumption. Smith et al. assessed the impact of COVID-19 pandemic on fossil fuel consumption and they anticipated that despite the reduction in the consumption during the pandemic, there will be a robust growth in energy consumption after pandemic, especially for emerging countries (Smith et al. 2021). Wang and Zhang indicated that China’s economic growth has a significant impact on energy consumption of high-income countries (Wang and Zhang 2021). Their results are given in Table 5:

**Table 5:** Energy consumption with respect to income of countries

Category of investigated countries	High income	Upper middle income	Lower middle income
Grow in energy consumption (%)	0.11–0.45	0.08–0.33	0.02–0.05

Industrialization, urbanization and economic growth of developing or least developed countries leads to a peak of energy demand in the world (Jiang and Lin 2012). The use of fossil fuels got increased up to 98% of total demand of energy in some countries (Perea-Moreno et al. 2016). The increasing demand of hydrocarbon energy and its usage restriction lead oil-producing countries to use of their potential to produce more oil and get more shares in oil market.  In the other words, the significance of EOR operations is increasing in recent years.

In a comprehensive evaluation, economic assessment in oil industry results in determining whether extraction and EOR operations are commercially efficient to develop an oilfield. The EOR processes are the efforts of energy industry beyond the conventional exploration and production strategies which are more dependent to technology than geography or geology. There are limited studies which investigated economics of EOR projects. Bondor examined how economic analysis can be used to determine the most effective direction for research. He found that economic analysis determines the fundamental limitation process which preclude the practical process (Bondor 1993). Flanders  and investigated the economic feasibility of performing CO_2_ EOR operation in small- and medium-size fields. They found that the EOR tax incentives reduces the risk of undertaken CO_2_ project and the economic feasibility of CO_2_ EOR is very field-specific (Flanders and McGinnis 1993). Zekri and Jebri applied economic sensitivity analysis on key variables such as oil prices, the price of injection solvent, capital expenditures, operating expenses and oil recovery to develop sensitivity graphs for each variable to assess future engineering EOR planning. They applied this empirical analysis for Libyan oil reserves. Their preliminary investigation indicate that the techniques of chemical EOR process are not cost-effective due to the logistics of supplying large volume of chemicals (Zekri and Jerbi 2002).

**Fig. 9:**
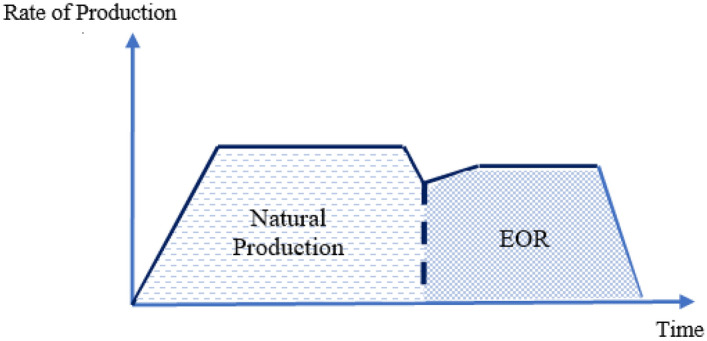
Oil production rate from a petroleum reservoir versus time for production under natural mechanisms and EOR process

According to regular production function, the rate of production (marginal production) from oil reservoirs varies along the stage of production, as shown in Fig. 9. In the beginning of production, the output rises in an increasing rate, then the rate of production constant for a long duration. Subsequently, the rate of production decreases and the producer has to decide among: (1) continuing the production to reach the zero rate of production, (2) abandoning the field or (3) starting the EOR operation. As illustrated in Fig. 9, by applying EOR operations the rate of production would increase. Then the production increases in a constant rate that is lower than the latter constant rate of conventional production period. Finally, the production would crash sharply.

**Fig. 10:**
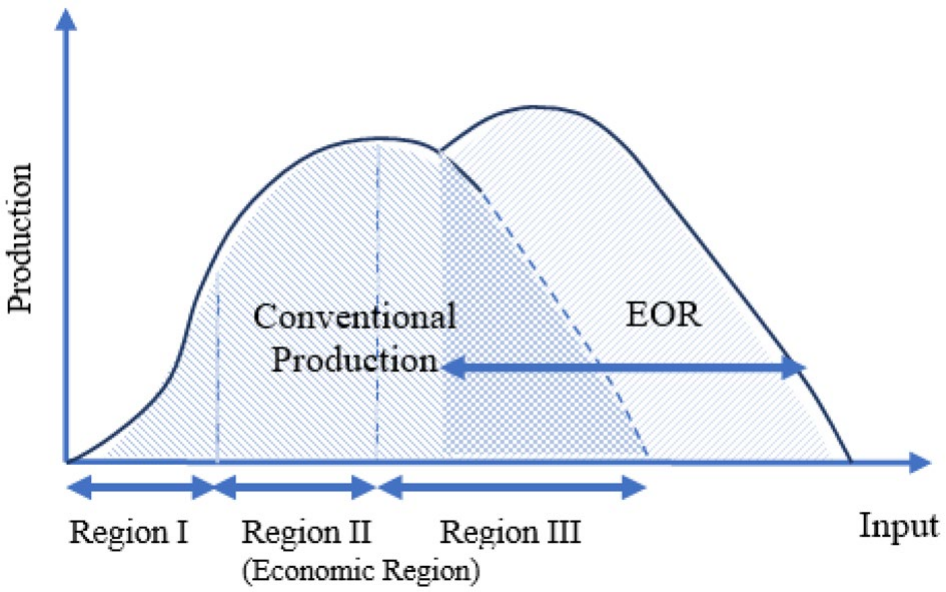
Oil production during different economic regions

Figure 9 indicates the output corresponding to production function in Fig. 10. The conventional stages of production function are illustrated in Fig. 10. In the first region of production, the ratio of change in output to the variation of input is greater than 1 (increasing return to scale). In the second region which called economic region, the ratio is positive and less than 1 (constant and diminishing return to scale). The economic region continues to the maximum point of accumulative production. Then the third region begins where the ratio is negative (decreasing return to scale). Conventionally, the producer may decide to cease the production in third region, though beginning the EOR operation can be an option. The mentioned ratio (change of output to the variation of input) for EOR operation is lower than economic region in conventional production. In the following an economic model is introduced to find out the optimum point of third region for beginning the EOR operations. The optimal amount of production is the other parameter which could be determined by the  aforementioned model.

#### Hotelling evaluation principle

Swierzbinski argue that Hotelling evaluation principle is an economic approach to consider the choice of extraction of exhaustible resource as an investment decision (Swierzbinski, 2013). Jamal and Crain applied Hotelling evaluation principle to calculate the net value of an exhaustible natural resource (Jamal and Crain 1997). The cost increases at the prevailing interest rate. This expectation is due to intertemporal maximization by the owner of resource. Miller and Upton used Hotelling evaluation principle conducted some analysis on optimal patterns of economic assessment for an exhaustible resource. They applied Eq. 4 to optimize the net value:

4 $${\text{Max}} V_{0} = \mathop \sum \limits_{t = 0}^{N} \frac{{P_{t} q_{t} - C_{t} \left( {q_{t} , Q_{t} } \right)}}{{\left( {1 + r} \right)^{t} }}\quad {\text{Subject to}}\quad q_{t} \le R_{0}$$

where ${P}_{t}$ denotes market price, which is determined in a competitive market. ${q}_{t}$ represents the amount of extraction at time *t*, and *r* denotes prevailing interest rate. *N* is the abandonment time for exhaustible resources, and ${R}_{0}$ denotes total reserves. It is assumed that there is no uncertainty in prevailing interest rate during the time of investment. ${C}_{t}$ is the cost of extraction, which is a function of ${q}_{t}$ and accumulative amount of production at a specified time interval (${Q}_{t}$). The accumulative amount of production is calculated by Eq. 5.

5 $$Q_{t} = \mathop \sum \limits_{s = 0}^{t} q_{s} ,$$

where ${q}_{s}$ is the amount of produced oil at time s (Miller and Upton 1985). With respect to economic fundamentals, the cost of production increases trivially by enhancing the production. Therefore, the amount of partial derivative $\left( {\delta C_{t} } \right)/\left( {\delta q_{t} } \right)$ should be positive during lifetime of oil reservoir. The derivative of $\left( {\delta C_{t} } \right)/\left( {\delta Q_{t} } \right)$ is nonnegative and its magnitude will increase by EOR process. The first-order condition for profit optimization in each period is:

6 $$\left( {p_{t} - c_{t} } \right)\left( {\frac{1}{1 + r}} \right)^{t} - \mathop \sum \limits_{s = t}^{N} \left( {\frac{{\delta C_{s} }}{{\delta Q_{s} }}} \right)\left( {\frac{1}{1 + r}} \right)^{s} = \lambda , \quad t = 0, \ldots , N$$

where $\lambda$ represent Lagrange multiplier. For simplicity, it is assumed that $\delta {C}_{s}/\delta {Q}_{s}=0$; therefore,

7 $$\left( {p_{t} - c_{t} } \right)\left( {\frac{1}{1 + r}} \right)^{t} = \lambda$$

By solving the system of difference equation, we obtain familiar Hotelling evaluation principle:

8 $$\left( {p_{t} - c_{t} } \right) = \left( {p_{0} - c_{0} } \right)\left( {1 + r} \right)^{t}$$

Based on Eq. 8, the efficient intertemporal production of an exhaustible resource is a function of net value of product, which grows over time at the real rate of interest. Note that Reynolds argues that Hotelling evaluation principle is an appropriate model to investigate the economic limits for production from oil and gas fields. Hotelling principle is progressed and developed by several researchers in recent years (Reynolds 2013). Slade and Thille developed the model by considering the role of oil as a risky asset in financial market (Slade and Thille 1997). In the following, we abandon several assumptions which are accounted in model of Miller and Upton. Hotelling evaluation principle could be simplified by assuming constant return to scale,^8^ which yield:

9 $$V_{0} = \left( {p_{0} - c_{0} } \right)\mathop \sum \limits_{t = 0}^{N} q_{t} = \left( {p_{0} - c_{0} } \right)R_{0} ,$$

Equation 9 reveals that the value of total reserve ($${R}_{0})$$ depends on net value of each produced oil barrel. At the start of EOR procedures, diminishing return to scale^9^ is inevitable. Therefore, the derivative $$\delta C\_t/\delta q\_t$$ and $${\delta }^{2}{C}_{t}/\delta {{q}_{t}}^{2}$$ is positive for secondary and tertiary (EOR) production. To investigate production under diminishing return to scale condition, Eq. 8 is transformed to:

10 $$V_{0} = \mathop \sum \limits_{t = 0}^{N} \left( {p_{0} - c_{0} } \right)q_{t} \left( {\frac{1}{1 + r}} \right)^{t} - \mathop \sum \limits_{t = 0}^{N} F_{t} \left( {\frac{1}{1 + r}} \right)^{t} ,$$

where ${F}_{t}$ is the difference between average and marginal cost. In general form:

11 $$V_{0} = \left( {p_{0} - c_{0} } \right)R_{0} - \mathop \sum \limits_{t = 0}^{N} F_{t} \left( {\frac{1}{1 + r}} \right)^{t} ,$$

The simplification assumption of $\delta {C}_{s}/\delta {Q}_{s}=0$ is abandoned due to inflationary conditions that most major developing oil-producing countries are encountered. The additional term is a constant. By substituting the first-order conditions in Eq. 11:

12 $$V_{0} = \lambda \mathop \sum \limits_{t = 0}^{N} q_{t} + \mathop \sum \limits_{t = 0}^{N} \mathop \sum \limits_{s = t}^{N} \left( {\frac{{\delta C_{s} }}{{\delta Q_{s} }}} \right)q_{t} \left( {\frac{1}{1 + r}} \right)^{s} - \mathop \sum \limits_{t = 0}^{N} F_{t} \left( {\frac{1}{1 + r}} \right)^{t} ,$$

where

13 $$\lambda = \left( {p_{0} - c_{0} } \right) - \mathop \sum \limits_{s = 0}^{N} \left( {\frac{{\delta C_{s} }}{{\delta Q_{s} }}} \right)\left( {\frac{1}{1 + r}} \right)^{s} .$$

by substituting $\lambda$ in 12, Eq. 14 will be achieved:

14 $$V_{0} = \left( {p_{0} - c_{0} } \right)R_{0} - \mathop \sum \limits_{t = 1}^{N} \mathop \sum \limits_{s = 0}^{t - 1} \left( {\frac{{\delta C_{s} }}{{\delta Q_{s} }}} \right)q_{t} \left( {\frac{1}{1 + r}} \right)^{s} - \mathop \sum \limits_{t = 0}^{N} F_{t} \left( {\frac{1}{1 + r}} \right)^{t} .$$

The last two expressions are constant and both of them are nonnegative. To determine the proper enhancement oil recovery operation, these two expressions should be considered for each well by its engineering parameters. Empirically while the EOR operation is based on application of nanoparticles, the revenues (outputs) and costs (inputs) for the model are tabulated in Table 6:

**Table 6:** Revenues (outputs) and costs (inputs) which should added to the model

Inputs	Output
Drilling and completion of injection wells (if needed)	Crude oil due to application of nanoparticles
Study, evaluation and simulation costs	
Supplement of nanoparticles	
Cost of stabilization process	
Cost of water treatment for preparing nanofluid	
Cost of injection equipment (pumping, pipelines, etc.)	
Cost of human resources (wages)	
Providing separation equipment to separate nanoparticles from produced oil	
Drilling and completion of new production wells (if needed)	
Other costs of production under new conditions	
Maintenance of wellhead equipment	

Consequently, after the peak of production of the well, two choices are to conserve remaining reserves or doing enhancement oil recovery operation. For both, there is uncertainty about technology and less resource lose social value which is irreversible sunk cost (related to uncertainty). In this regard, applying the engineering parameters consistent with each well properties removes uncertainties and reduce sunk cost that make Hotelling evaluation principle available and more precisely to use.

### Conclusions

This review represented an insight into application of nanotechnology for EOR intends from the prospective view of a petroleum engineer. Based on valuable results achieved by various researchers and scientific theories, some important points could be concluded. The conclusion could be summarized as below:

- Although stability of nanofluids in reservoir condition is a challenge, there is numerous benefits for application of NPs through EOR process.
- Nanoparticles have the potential to alter the state of wettability of formation rock by creating a new surface. They could be adsorbed to the surface of rock by precipitation (due to gravity) and electrostatic force (due to difference charge of NPs and rock surface).
- NPs usually have tendency to move forward to the interface of oil and water. This tendency and their activity at the interface lead to IFT reduction.
- Catalytic effect of NPs and adsorption of asphaltene content, prevents asphaltene deposition and, respectively, reduces the viscosity of heavy oils.
- Application of high-pressure homogenizer is the most effective physical method for stabilizing nanofluids. However, chemical methods reflect better response in comparison with physical method.
- Application of surfactants and polymers and pH control is the most common chemical stabilization processes.
- Hybrid application of NPs and surfactant enhances the efficiency of NPs by adsorption of surfactant micelles and gradual desorption. In addition, lowering the retention of surfactants in porous media alongside with improved stability of NPs enhances the amount of recovered oil.
- Hybrid application of NPs with foams increases foam stability and amend sweep efficiency.
- Hybrid application of NPs with polymers is an effective method for increasing the strength of polymer solutions.
- Hybrid application of NPs with low-salinity water empowers wettability alteration under two main mechanisms. Low-salinity water creates a new surface on the rock by dissolution and hydration of minerals. Besides, subsidence of NPs on the surface of rock with gravity precipitation and electrostatic adsorption covers the surface.
- Hotelling method represents an appropriate model for economic evaluation of EOR process.

## List of symbols

*A*: Hamaker constant (J)
*C*: Volumetric concentration (weight/vol)
${C}_{s}$: Cost of extraction at time *s* (currency)
${C}_{t}$: Cost of extraction (currency)
${C}_{0}$: Cost of extraction at time 0 (currency)
*E*: Adhesion energy (KBT)
*E*_0_: Strength of electrical field (V/m)
${F}_{t}$: The difference between average and marginal cost (currency)
*K*_0_: Solvation constant (dimensionless)
*Μ*: Electrophoretic mobility (μm Cm/v s)
*N*: Abandonment time for exhaustible resources (day, month, year)
${\rm P}$_0_: Market price at time 0 (currency)
${P}_{t}$: Market price at time $t$ (currency)
${Q}_{t}$: Cumulative amount of production (Bbl)
${Q}_{s}$: Cumulative amount of production at time *s* (Bbl)
${q}_{s}$: Amount of produced oil at various time *s* (Bbl)
${q}_{t}$: Amount of extraction at time *t* (Bbl or ft3)
$r$: Prevailing interest rate (currency)
Rel. perm: Relative permeability (dimensionless)
${R}_{0}$: Total reserves (Bbl)
R.F: Recovery factor (%)
*S*: Saturation (dimensionless)
*t*: Time (S, h, day)
*V*: Shape factor of dispersed particle (Dimensionless)
${V}_{0}$: Net value (currency)
$$\Theta$$: Contact angle (°)
$\lambda$: Lagrange multiplier (dimensionless)
${\mu }_{r}$: The ratio of dispersion viscosity to bulk phase viscosity (dimensionless)
${\varvec{v}}$: Velocity of particles (m/s)
${\sigma }_{12}$: Interfacial tension (mN/m)
*Φ*: Porosity (dimensionless)

## Abbreviation

AFM: Atomic force microscopy
AOS: Alpha olefin sulfonate
CFD: Computational fluid dynamics
CMC: Critical micelle concentration
CTAB: Hexadecyltrimethylammonium bromide
D: Darcy
DLS: Dynamic light scattering
DLVO: Theory of Derjaguin, Landau, Verwey and Overbeek
EDX: Energy-dispersive X-ray
E-GO: Edge graphene oxide
EOR: Enhance oil recovery
FESEM: Field emission scanning electron microscopy
FTIR: Fourier transform infrared
GLYMO: (3-Glycidyloxypropyl) trimethoxysilane
GO: Graphene oxide
HPAM: Hydrolyzed polyacrylamide
IEP: Isoelectric point
IFT: Interfacial tension
IS: Ionic strength
kHz: Kilohertz
MoS_2_: Molybdenum disulfide
NPs: Nanoparticles
PAM: Polyacrylamide
PGN: Polymer grafted nanoparticles
pH: Potential of hydrogen
PNS: Hybrid of polymer nanofluid suspension
ppm: Part per million
Psi: Pound force per square inch
SBS: (Dimethyl(3-(trimethoxysilyl) propyl)-ammonio) propane-1-sulfonate
SDS: Sodium dodecyl sulfate
SEM: Scanning electron microscope
SurfaSil: C_8_H_24_Cl_2_O_3_Si_4_
S-GO: Surface graphene oxide
TEM: Transmission electron microscopes
TOC: Total organic carbon
UV: Ultraviolet
Wt%: Weight percent
XRD: X-ray diffraction

## Footnotes

1 Total organic carbon.

2 Ionic strength.

3 Total dissolved solids.

4 Interfacial shear viscosity is defined as the ratio between the shear stress and the shear rate in the plane of the interface.

5 Alpha olefin sulfonate.

6 Molybdenum disulfide.

7 Foam volume generated at the end of stirring stage where foam is generated.

8 Constant return to scale: proportional equality between changes of input and output ($\delta {C}_{t}/\delta {q}_{t}$=constant and ${\delta }^{2}{C}_{t}/\delta {{q}_{t}}^{2}\hspace{0.17em}$= 0).

9 Diminishing return to scale: increasing the input enhances the output by less ratio ($\delta {C}_{t}/\delta {q}_{t}$ and ${\delta }^{2}{C}_{t}/\delta {{q}_{t}}^{2}$ are positive).
